# CHB‐Induced Immune Zonation Chaos Elicited LXRα‐mediated Lipid Metabolism Disorders in Kupffer Cells to Induce Cancer Stem Cell Formation

**DOI:** 10.1002/advs.202510275

**Published:** 2025-10-30

**Authors:** Jingqi Shi, Qingyu Li, Jian Li, Jian Bai, Ji Xi, Qi He, Jianglin Zhou, Xuejun Wang, Xiang Song, Xiaoju Li, Xiangpei Yue, Xiaochang Zhang, Zhen Sun, Jiangbo Li, Wen Yang, Yuke Cui, Wenjie Shu, Liang Guo, Shengqi Wang

**Affiliations:** ^1^ Bioinformatics Center of AMMS Beijing 100850 P. R. China; ^2^ Beijing Institute of Basic Medical Sciences Beijing 100191 P. R. China

**Keywords:** chronic HBV, inflammation‐to‐carcinoma transition, Kupffer cell, lipid metabolism disorder, LXRα

## Abstract

Hepatic intercellular communication is the driving force for the progression of chronic Hepatitis B virus (CHB)‐associated hepatopathologies, with the dynamic molecular mechanisms largely unknown. Combining scRNA‐seq and spatial transcriptomic analysis, the kinetic landscape of the liver microenvironment across time and space in AAV‐HBV mice, which develop from inflammation to ultimately hepatocellular carcinoma is generated. Kupffer cells (KCs), originally resided within the peri‐portal area, are persistently recruited to the HBV‐enriched peri‐central region via increased CXCL9 produced by endothelial cells, facilitating the interaction between KCs and HBV^+^ hepatocytes to induce LXRα deficiency‐mediated lipid metabolism disorders (LMD) in KCs. In turn, KCs with LMD elicited cancer stem cell formation from HBV^+^ hepatocytes via Stat3 pathway, activated by the chemokine network within the crosstalk. Moreover, miR‐155‐mediated post‐transcriptional regulation and ASGR1‐dependent degradation collaboratively regulated LXRα downregulation in KCs. LXRα deficiency in KCs is also detected in the tumor tissues of HBV^+^ patients compared to that of the normal and tumor‐adjacent tissue. Importantly, LXRα upregulation in KCs constrained fibrosis and cancer stem cell formation. For the first time, the role of KC zonation in disease progression has been revealed, highlighting LXRα in KCs as a promising target for the early intervention in the transition from CHB‐induced inflammation to cancer.

## Graphical abstract

By profiling the spatiotemporal hepatic landscape of CHB mouse models, the originally peri‐portal localized KCs migrated to the peri‐central in a CXCL9‐CXCR3‐dependent manner, facilitating their interaction with HBV^+^ hepatocytes. The interaction promoted LMD in KCs through ASGR1‐induced LXRα degradation, which, in turn, induced CSC formation via Stat3 activation in hepatocytes. LXRα agonists constrained CHB‐induced fibrosis and CSC formation.

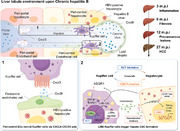

## 1 Introduction

Chronic Hepatitis B virus (CHB) infection affects ≈300 million individuals worldwide and is the leading cause for liver cirrhosis and hepatocellular carcinoma (HCC) worldwide,^[^ ^1^, ^2^ ^]^ inducing more than 884 000 deaths annually.^[^ ^2^, ^3^ ^]^ Multiple antiviral drugs have been developed to target the life cycle of Hepatitis B virus (HBV),^[^ ^4^ ^]^ however, the persistence of viral covalent closed circular DNA (cccDNA) and the defective immune response of the host challenge the therapeutic effects. Therefore, it is an urgent need for early interventions to halt, or even reverse, CHB‐induced inflammation‐to‐carcinoma transition (ICT).

HBV itself is suggested to be non‐cytopathic, as gene clusters between HBV‐infected and uninfected cells were similar.^[^ ^5^ ^]^ Therefore, CHB‐associated liver pathologies are considered to be mainly caused by long‐term hepatic inflammation.^[^ ^6^ ^]^ Liver is an immunologically complex organ with a diverse immune cell repertoire. Several groups have deciphered the hepatic immune landscape in healthy humans and mice, indicating the high heterogeneity of intrahepatic macrophages which represent the key component of liver microenvironment.^[^ ^7^, ^8^, ^9^, ^10^ ^]^ The alterations of liver immune under cirrhosis or HBV/HCV‐related HCC have also been explored, and liver macrophages been suggested to participate in fibrotic niche formation and tumor progression by its interplay with endothelial cells and T cells, respectively, highlighting the central role of macrophages in hepatic pathogenesis.^[^ ^11^, ^12^ ^]^

Kupffer cells (KCs), as a self‐maintaining and liver‐resident population of liver macrophages, modulate hepatic homeostasis and each pathological stage that also occurs in CHB‐associated diseases.^[^ ^13^, ^14^ ^]^ In turn, the phenotype and functions of KCs are shaped by the surrounding microenvironment. It has recently been revealed that fibrosis reduces the anti‐bacteria capacity of KCs by depriving their contract with parenchymal cells,^[^ ^15^ ^]^ underscoring the critical role of interactions between non‐parenchymal and parenchymal cells in modulating liver functions. Currently, the scenario of liver inflammatory microenvironment during the natural history of CHB‐related liver diseases remains unclear. Additionally, there has been limited exploration into the dynamics of KCs and their interplay with neighboring cells during the process.

Moreover, liver zonation is fundamental for proper functioning of the organ and has gained great attention. Hepatocytes within the lobule lining up along the porto‐central axis are divided into three zones to perform diverse functions,^[^ ^16^, ^17^, ^18^, ^19^ ^]^ and thus it is not surprising that the zonation of liver extends to liver‐resident immune cells. More recently, myeloid and lymphoid cells have been found to concentrate around the periportal region.^[^ ^20^, ^21^, ^22^, ^23^ ^]^ Moreover, the spatial polarization of immune cells have been proved to be efficient in protecting systemic bacteria dissemination, which implies that immune zonation is critical to host defense.^[^ ^20^ ^]^ However, the alteration of hepatic immune zonation and its role in promoting CHB progression have not been discovered yet.

In this study, we utilized 10X Genomics single‐cell sequencing (scRNA‐seq) and Visium spatial transcriptomic (ST) analysis to create, for the first time, a dynamic landscape of hepatic inflammatory microenvironment in an adeno‐associated virus‐delivered HBV (AAV‐HBV) mice model. This model naturally progresses from chronic inflammation to fibrosis and HCC over a period of 27 months post‐infection. Through this approach, we uncovered a novel “trilogy” mechanism of Kupffer cells during disease progression. During the long‐term HBV infection, KC zonation disturbance induced by increased chemokine (C‐X‐C motif) ligand 9 (CXCL9) within HBV‐enriched peri‐central area facilitated the interaction between macrophages and HBV^+^ hepatocytes, which elicited lipid metabolism disorder in KCs dominated by LXRα deficiency. In turn, lipid metabolism disordered KCs led to hepatic cancer stem cell (HCSC) formation via the chemokine network including chemokine (C‐C motif) ligand 19 (CCL19), CXCL10, and CXCL11 within the crosstalk. Thus, our study has shed new light on the temporal and spatial roles of KC in the inflammation‐to‐carcinoma transition and provided potential targets for the early interventions of CHB‐associated liver diseases.

## 2 Results

### 2.1 ScRNA‐Seq and Spatial Transcriptomic Analysis of Liver Inflammatory Microenvironment During the Progression of CHB‐Associated Liver Diseases

**Figure 1:**
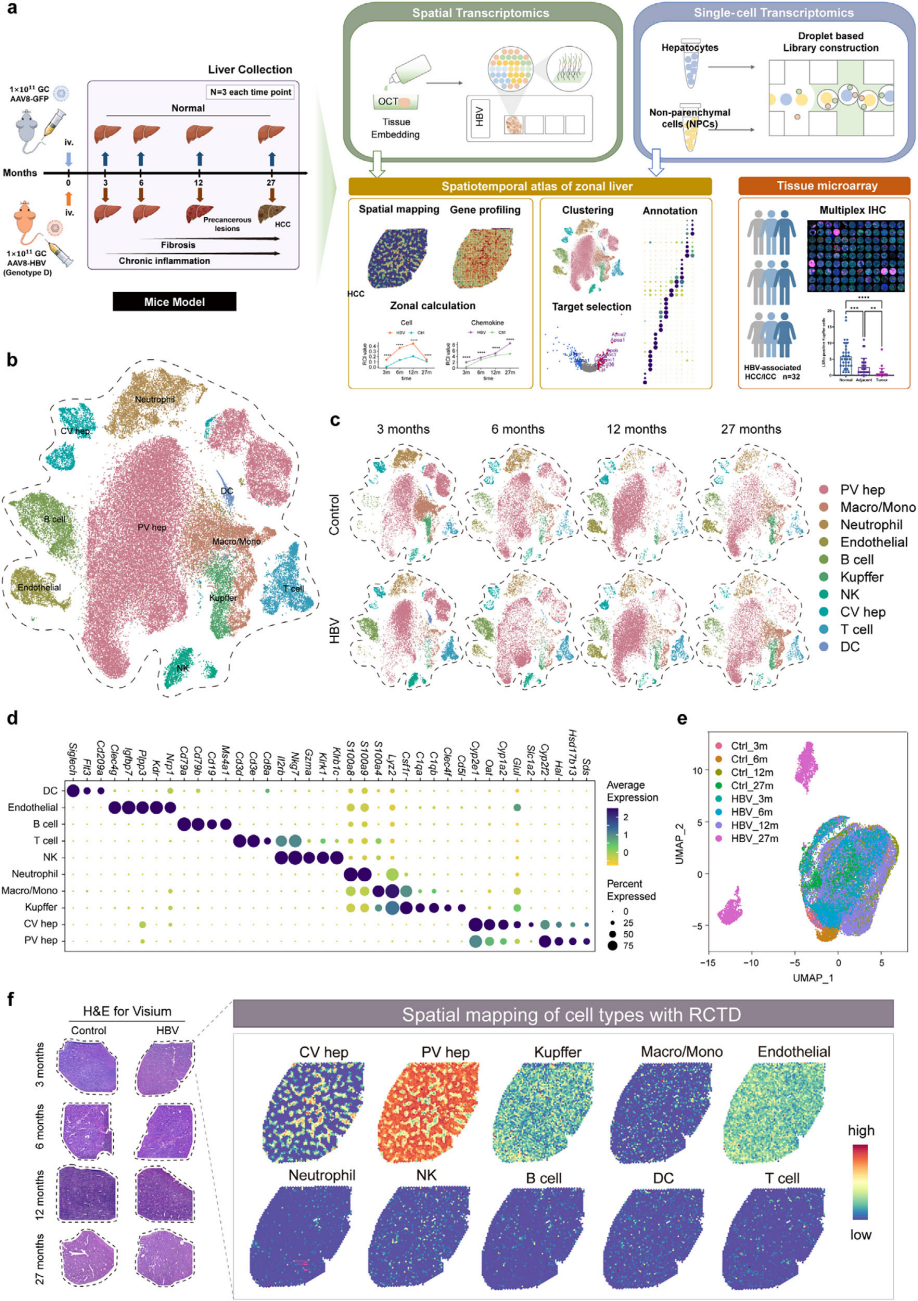
Dynamic scRNA‐seq and spatial transcriptomic analysis of liver microenvironment during the progression of CHB‐associated liver pathologies. a) The schematics of single cell isolation, liver tissue dissection, and analysis of the livers of CHB mice developing from chronic inflammation to fibrosis and cancer using 10X Genomics scRNA‐seq and Visium platform (*n* = 3). b) t‐SNE plot of scRNA‐seq data showing two clusters of hepatocytes and eight clusters belonging to the non‐parenchymal cells (NPCs) from the livers of control and CHB mice. Three mice livers from each group were dissected for hepatocytes isolation and three for NPCs at indicated time points. c) t‐SNE plot of scRNA‐seq data at four time points (3, 6, 12, and 27 mpi), respectively. d) Dot plot analysis using the known gene expression profiles of hepatocytes and immune cells. The identity of each cluster was assigned by matching the cluster expression profile with established cell‐specific signature genes expression. e) UMAP plot of the Visium spatial transcriptomic data of the livers derived from the control and CHB mice at different time points. The livers of the control and CHB mice at different time points (3, 6, 12, and 27 mpi) were subjected to 10X Visium spatial transcriptomic analysis. f) The representative images of Visium plot of the liver from control and CHB mice. The freshly frozen sections of the liver of the control and CHB mice were subjected to H&E staining, followed by 10X Visium transcriptomic analysis (left). Robust cell type decomposition (RCTD) was used to map the spatial distribution of certain cell types by integrating scRNA‐seq and spatial transcriptomic data on the Visium plots of the liver tissue (right). mpi, month postinfection.

We aimed to dynamically profile the liver microenvironment upon persistent CHB infection across time and space. This was achieved by combining 10X Genomics scRNA‐seq of both the hepatocytes and non‐parenchymal cells with Visium ST analysis of the freshly frozen liver specimens from the control and CHB models undergoing different stages of hepatopathologies. Additionally, in our study, we further analyzed the liver tissue microarray of HBV‐positive hepatocellular carcinoma (HCC) patients (**Figure** **1**a).

As AAV‐HBV mouse models naturally progress from chronic inflammation to fibrosis/cirrhosis and HCC,^[^ ^24^, ^25^, ^26^, ^27^ ^]^ we used AAV8‐mediated delivery of 1.3 × HBV genome (genotype D) to develop a chronic HBV mouse model. In this model, persistent infection of HBV was marked by a high serum HBV DNA content that begun to rise as early as one week post‐infection (p.i.) and maintained above 1×10^5^ IU/ml throughout the entire duration of the 108 weeks (27 months), accompanied by a consistent detection of Hepatitis B surface Antigen (HBsAg) and Hepatitis B e Antigen (HBeAg) in mouse sera. HBsAg was detectable 4 weeks p.i. with a peak at 38 weeks p.i. at ≈27 000 ng mL^−1^, and then declined to a plateau at ≈3000 ng mL^−1^ until 72 weeks p.i. HBeAg started to increase as early as one‐week p.i. and maintained above 1000 NCU/ml until 72 weeks p.i. (Figure S1a, Supporting Information). The body weight of AAV‐HBV model mice was also decreased (Figure S1b, Supporting Information).

After 24 weeks (6 months) of chronic infection, sporadic collagen fibers began to be detected within the liver, with an upregulation of *matrix metalloproteinase (Mmp)‐2* (fibrosis‐associated marker genes). Severe fibrosis occurred 12 months p.i. (mpi), evidenced by an obvious collagen fiber staining and a dramatic elevation of marker genes (Figure S1c, Supporting Information). Additionally, the serum concentration of alpha‐fetoprotein (AFP), one of the most widely used diagnostic biomarkers for HCC, was significantly increased in the 12‐month of CHB models, with small cell change (SCC, a characteristic of early precancerous lesions of HCC)^[^ ^28^, ^29^ ^]^ appeared in the liver sections (Figure S1d,e, Supporting Information), which indicated the precancerous lesion. At 27 mpi., along with severe fibrosis, HCC occurrence was verified by immunohistochemistry (Figure S1f, Supporting Information).

To unveil the dynamic profile of liver inflammation upon CHB infection throughout the whole process, the livers of the control and AAV‐HBV mice from the indicated time points (3, 6, 12, and 27 mpi) were collected for parenchymal and non‐parenchymal single cell dissociation, followed by 10X Genomics scRNA‐seq analysis. After removing the doublets and cells with poor quality (high mitochondrial gene expression or low genes detected) (Figure S1g,h, Supporting Information), the remained single cells were classified into two clusters of hepatocytes and eight clusters of NPCs based on the expression of unique signature genes (Figure 1b–d; Figure S1i, Supporting Information), delineating the high diversity of liver microenvironment during disease progression. Moreover, the livers of the control and AAV‐HBV mice taken from different time points of post‐infections were also subjected to spatial transcriptomic (ST) analysis, with quality control and dimensional reduction and clustering done (Figure 1e,f; Figure S1j, Supporting Information). The uniform manifold approximation and projection (UMAP) plot showed that the ST spots of liver tissue from the 27‐month of mice were clustered separately from the other samples, indicating an obvious phenotype change of the liver in cancerous stage, compared to that of non‐cancerous phases (Figure 1e). Additionally, in the 27‐month of ST data, *AFP*‐expressed spots were significantly enriched, evidencing the occurrence of HCC (Figure S1k, Supporting Information).

Taken together, the AAV‐HBV mouse model we developed here successfully progressed from chronic inflammation into fibrosis and carcinoma upon long‐term infection. Based on this model, we profiled the dynamic single‐cell transcriptomics of the liver inflammatory microenvironment across time and space by combining scRNA‐seq and ST analysis of liver specimens from different pathological stages during CHB infection.

### 2.2 KCs were Recruited to HBV‐Enriched Peri‐Central Area by CXCL9 to Facilitate Their Crosstalk with HBV^+^ Hepatocytes

**Figure 2:**
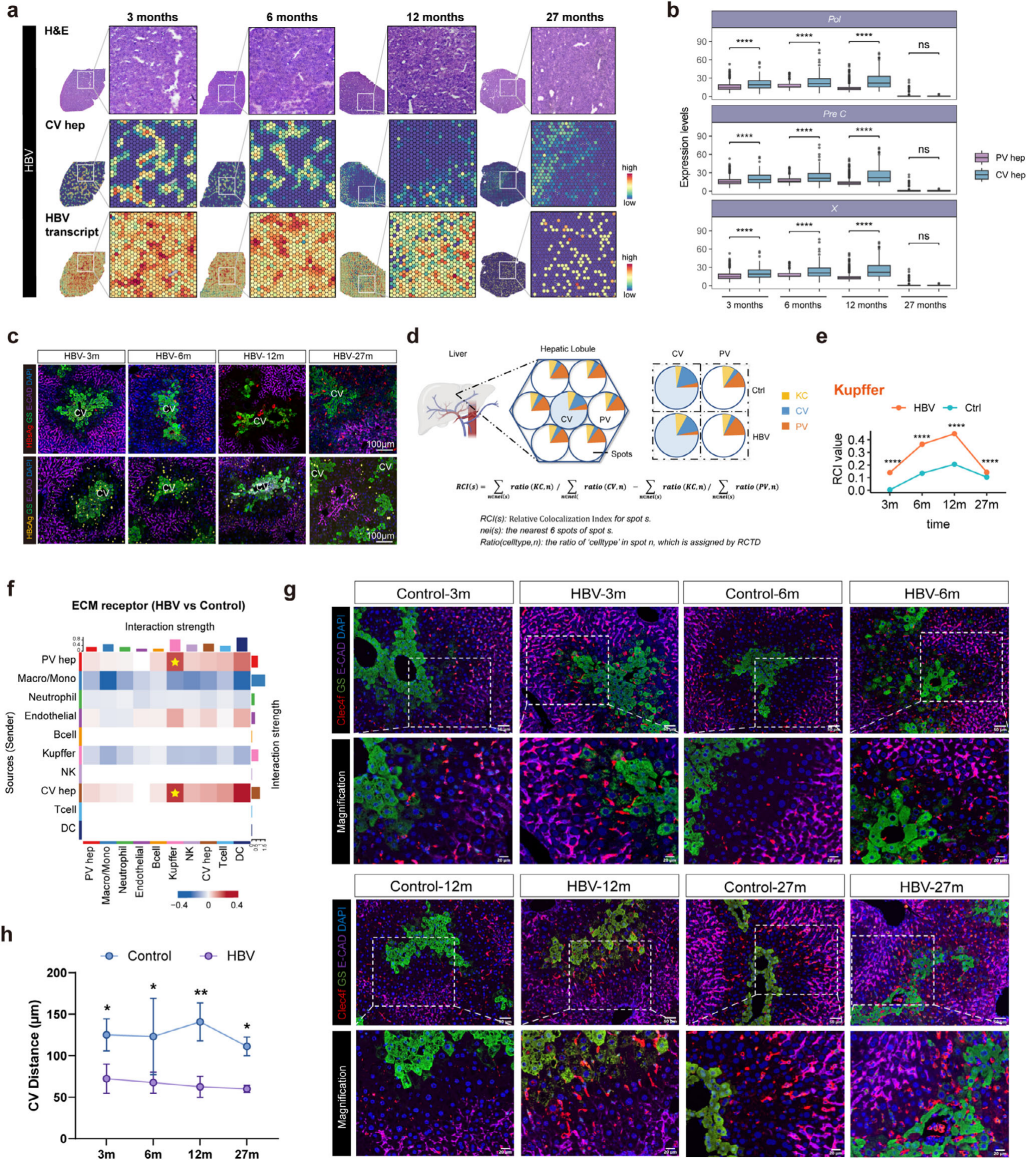
The spatial distribution of Kupffer cells (KCs) from peri‐portal toward peri‐central region within the lobule under long‐term HBV infection. a) The distribution of KCs and HBV transcript on the Visium Plots of the livers. The localization of CV hepatocytes (CV hep, upper) and HBV transcript on the Visium plots of the livers of CHB models at indicated time points were analyzed by RCTD. b) The expression of HBV transcripts in CV hepatocytes and PV hepatocytes of CHB mice at different time points. The expression of HBV transcripts (*Pol*, *Pre C*, and *X*) were significantly higher in CV hepatocytes (CV hep) than in PV hepatocytes (PV hep) at 3, 6, and 12 months post infection. c) Representative multiplex immunofluorescent (mIF) images of HBsAg (red), HBeAg (yellow), glutamine synthetase (GS)‐positive peri‐central region (green), and E‐cadherin‐positive peri‐portal zone (purple) on FFPE sections of the livers of the CHB mice at 3, 6, 12, and 27 mpi. d) The algorithm schematics of relative colocalization index (RCI). RCI was generated to quantify the spatial alteration of the immune cells. To quantify the zonation disturbance of KCs, first, RCTD analysis was used to estimate the proportion of Kupffer cells, peri‐central hepatocytes, and peri‐portal hepatocytes in each spot within the lobule, respectively. Second, the value of RCI was calculated, which was positively related to the abundance of KCs within the CV hepatocytes‐containing spots. e) RCIs for KCs of the control and HBV‐infected mice at indicated time points. The dynamic zonation variations of KCs from the control and CHB mice at indicated time points were examined by RCI values. f) Cell‐cell interaction analysis of different types of cells within the hepatic microenvironment. Hepatocytes, as senders, were highly interacted with Kupffer cells in HBV mice models based on cellular communication network analysis by cellchat. g) The dynamic spatial distribution of KCs during the progression of CHB‐associated pathologies. Multiplex immunofluorescent analysis of KC (Clec4f^+^, red), peri‐central area (GS^+^, green), peri‐portal area (E‐cadherin^+^, purple) and DAPI (blue) on FFPE slides of livers harvested at indicated time points from both control and HBV mice. h) Statistical analysis of the distance between KCs and central vein (CV) area upon long‐term of CHB infection. The average distance of individual KCs to the nearest central vein (CV Distance) was examined on three selected field‐of‐view of the mIF images from each group at indicated time points. (*n* = 3 biological independent samples). Data shown are mean ± SEM; *p<0.05, **p<0.01, ***p<0.001, ****p<0.0001.

To profile the inflammatory single‐cell transcriptome with liver morphological context, the spatial transcriptomics (ST) data was integrated with scRNA‐seq data through robust cell type decomposition (RCTD)^30^ ^]^ The marker genes for peri‐central vein (CV) hepatocytes were used to define liver zonation on the Visium plots (**Figure** **2**a). Given the potential link of HBV infection with liver terrain, the ST data were then aligned to HBV reference genome, identifying that the spots containing HBV transcripts were predominantly enriched in central vein (CV) hepatocytes (CV hep)‐containing spots at 3 and 6 mpi, while began to be detected outside of the CV hep spots at 12 mpi (Figure 2a). Additionally, scRNA‐seq data also revealed an enrichment of HBV transcripts in CV hepatocytes before HCC occurrence (Figure 2b). In the 27‐month models, HBV mRNA transcripts were found to be dispersed within the lobule (Figure 2a). Concomitantly, more HBcAg and HBsAg were stained within glutamine synthetase (GS)‐positive CV region than E‐cadherin‐positive portal vein (PV) area in the liver sections of the CHB mice at 3, 6, and 12 mpi by multiplex immunofluorescence (mIF) analysis, whereas after 27 mpi, the antigens were present throughout the lobule without a distribution bias (Figure 2c). Of note, the borders of the peri‐portal and peri‐central area within the lobule became less clear, and the mid‐zone between the CV and PV region was compressed, indicating the zoning of normal liver was interrupted with the progression of CHB (Figure 2c).

Since no data has been published regarding the changes of hepatic immune zonation or their potential role in CHB progression yet, we analyzed the localization of major immune cell types at indicated time points by ST data. To dissect the immune cell type with the most varied spatial distribution, we developed an index called relative colocalization index (RCI) to compare the proportion of different cell types within the spots along the porto‐central axis in a lobule, with the value of RCI positively related to the proximity of given cells to CV region (Figure 2d). Among all the immune cell types identified in scRNA‐seq, KCs exhibited the most significantly varied value of RCI (Figure 2e; Figure S2a, Supporting Information) and the alteration of KC RCI was consistent to the spatial changing pattern of HBV mRNA transcripts (Figure 2a–c). Briefly, more KC spots were detected in close proximity to the spots of CV hep in the Visium plots of CHB mice and thus the original peri‐portal zonation of KCs was disturbed, indicating that the KCs of CHB mice preferentially localized within HBV‐enriched CV region (Figure 2d,e; Figure S2b, Supporting Information). Notably, in ST data from the mice at 27 mpi, the RCI of KCs in CHB mice was reduced, which was probably attributed to the scattered expression of HBV mRNA transcripts and the disappearing hepatocyte zoning within the lobule (Figure 2a–c). Moreover, using the cellular communication network analysis, KC was also represented as the most active immune cell type to communicate with hepatocytes in all the infection time points of CHB mice, with a much higher interaction with CV hepatocytes than PV hepatocytes in 6 mpi, indicating the critical role of KCs in interacting with hepatocytes during the disease progression (Figure 2f; Figure S2c, Supporting Information). A more straightforward visualization of KC migrating from peri‐portal to peri‐central area was observed by mIF (Figure 2g). We measured distances in ImageJ using scale‐calibrated images. Specifically, the minimal distance from Kupffer cells (KCs, red) to the nearest central vein (CV, green) or portal vein (PV, purple) was determined. This quantifies KC proximity to peri‐central regions (red‐green distance) and peri‐portal regions (red‐purple distance). The average distance of individual KCs to the nearest CV (CV‐dis) and PV (PV‐dis) was measured on three selected field‐of‐view of the representative mIF images from each group, with CV‐dis significantly decreased and PV‐dis/CV‐dis increased in the AAV‐HBV mice (Figure 2g,h; Figure S2d,e, Supporting Information).

**Figure 3:**
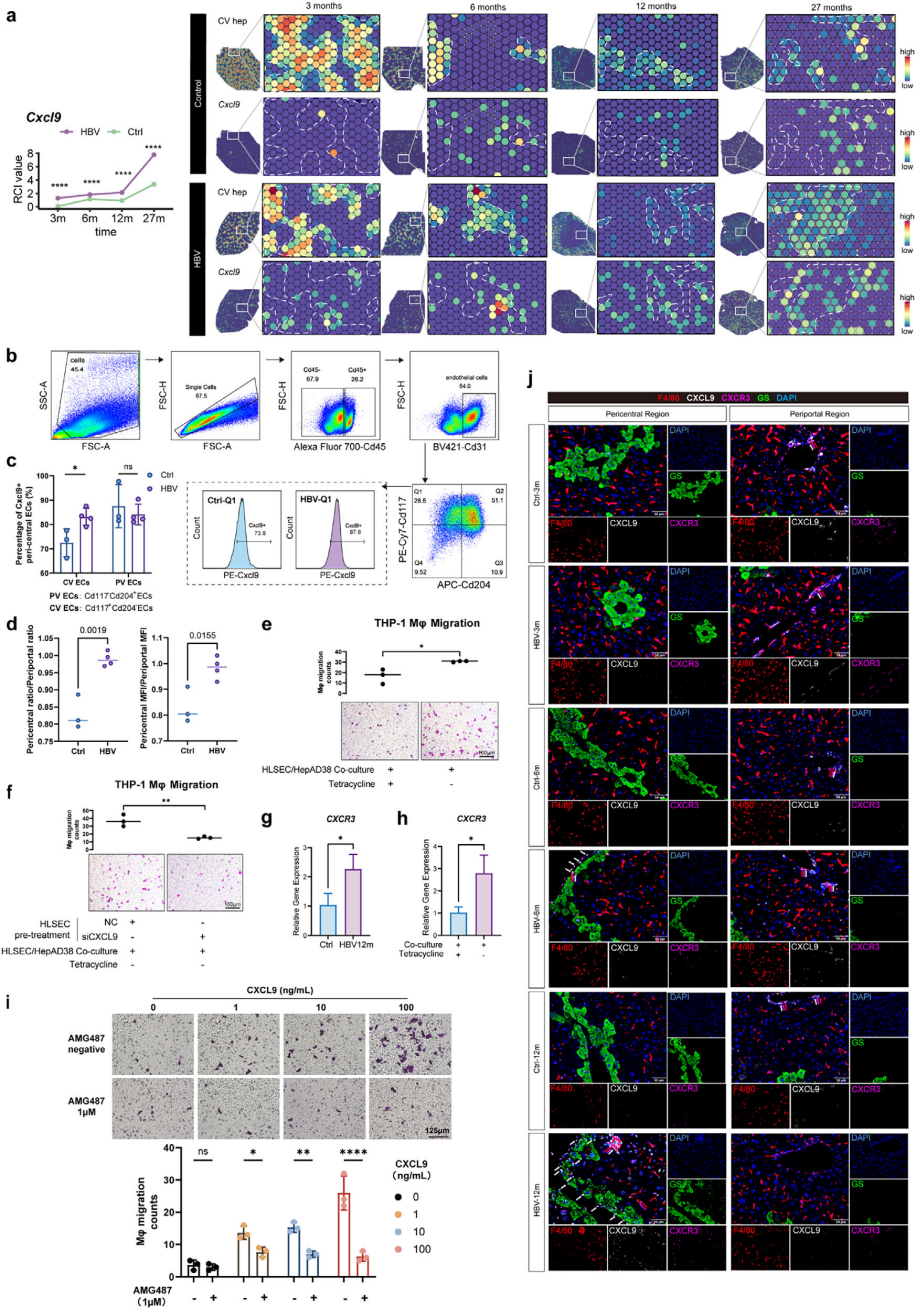
Increased CXCL9 secretion by endothelial cells within the peri‐central region was responsible for KC zonation alteration upon CHB infection. a) Persistently increased RCI for *CXCL9* throughout the whole experiment. Based on ST data, the RCIs for *CXCL9* of mice from the control and CHB mice at indicated times pi were examined (left). Representative images of the Visium plots displaying the peri‐central area delimited by white borders and *CXCL9*‐expressing spots within the same selected region of each liver specimen from the control and HBV mice at 3, 6, 12, and 27 mpi (right). b–d) The expression of CXCL9 in the endothelial cells of the peri‐central and peri‐portal region in the control and CHB mice models after 6 months of infection. The expression of Cxcl9 in the peri‐central ECs (CD45^+^CD31^+^CD117^+^CD204^‐^) and peri‐portal ECs (CD45^+^CD31^+^CD117^‐^CD204^+^) were analyzed by flow cytometry. The gating strategy of flow cytometry was shown (b). The percentage of Cxcl9^+^ peri‐central ECs was increased in CHB mice models (c). The ratio of the percentage of CXCL9^+^ ECs in the peri‐central ECs to that of the peri‐portal ECs, as well as the ratio of mean fluorescent intensity (MFI) of Cxcl9 of the peri‐central ECs to that of the peri‐portal ECs, was analyzed (d). e) Migration assay of THP‐1‐derived macrophages (Mφ) co‐cultured with HepAD38‐treated HLSEC. After a 48 h co‐culture with HepAD38 cells (tet^‐^ or tet^+^), HLSEC were seeded in the lower wells of the Transwell plate where THP‐derived macrophages were seeded in the upper chambers to allow another 48 h of co‐culture, followed by Sulforhodamine B (SRB) staining of macrophages migrating through the membrane. The experiments were performed in triplicate (*n* = 3). f) Knockdown of *CXCL9* by siRNA in HLSEC reduced the enhanced migratory capacity of macrophages induced by HepAD38/tet^‐^‐treated HLESCs. HepAD38/tet^‐^‐treated HLSECs were pre‐treated with *CXCL9* siRNA and then co‐cultured with THP‐derived macrophages for 48 h. SRB assay was used to stain the migrating macrophages. The experiments were performed in triplicate (*n* = 3). (g,h) The mRNA level of *CXCR3* in KCs from the 12‐month control and CHB mice (*n* = 3, g) and in THP‐derived macrophages h) co‐cultured with HepAD38 cells (tet^‐^ or tet^+^). The experiments were performed in triplicate (*n* = 3). i) Mφ were plated in the upper chamber of a Transwell system, while a gradient of concentrations of CXCL9 (0, 1, 10, 100 ng mL^−1^) was added to the lower chamber. Mφ were pre‐treated with the CXCR3 antagonist AMG487 (1 µM) for 24 h. SRB assay was used to stain the migrating macrophages. j) Multiplex IF analysis of KCs (F4/80, red), peri‐central area (GS, green), CXCL9 (grey), CXCR3 (purple), and DAPI (blue) on the liver FFPE sections from control and CHB mice of each time point. Compared to the control mice, CXCR3‐increased KCs in CHB models were recruited to the peri‐central area where an elevation of CXCL9 was also detected. Data are shown as mean ± SEM; *p<0.05, **p<0.01, ***p<0.001, ****p<0.0001.

Furthermore, the molecular motivation for KC migration was investigated. By analyzing the RCI of KC‐recruiting cytokines and chemokines based on ST data, we found that *CXCL9* in CHB mice exhibited significantly higher RCI than that of the control, with a concomitantly increased expression of *Cxcl9* spots within the CV area (the border of CV zonation was delimited in the Visium plots), which indicated that, in CHB mice, the production of CXCL9 in the HBV‐enriched peri‐central area was enhanced (**Figure** **3**a; Figure S3a, Supporting Information). CXCL9 could also regulate the recruitment of KCs in transgenic mouse. Using the GeoMx Digital Spatial Profiler (DSP), we explored the spatial distribution and transcriptome within peri‐portal (Zone 1) and peri‐central (Zone 3) regions of interest (ROIs) in fibrotic livers from transgenic mice. For each zone (1 and 3), *Cxcl9* expression levels were compared between transgenic and control mice. The bar plot revealed highly elevated *Cxcl9* expression, specifically within Zone 3, in transgenic mice (Figure S3b, Supporting Information). This finding was consistent with results observed in AAV‐HBV mice. *CXCL9* was proved to be expressed only in endothelial cells by scRNA‐seq data (Figure S3c,d, Supporting Information). It has been recently revealed that endothelial cell (EC)‐derived CXCL9 is critical for the peri‐central localization of KCs in the normal liver,^[^ ^20^ ^]^ which prompted us to focus on the potential change of the gradient of CXCL9 along the porto‐central axis upon CHB. In comparison to the control mice, the count and percentage of CXCL9^+^ peri‐central ECs (CD45^+^CD31^+^CD117^+^CD204^−^CXCL9^+^) were increased in the livers of the 6‐month CHB mice (Figure 3b,c). Additionally, the ratio of the mean fluorescent intensity (MFI) of CXCL9 of the peri‐central ECs to that of the peri‐portal (CD45^+^CD31^+^CD117^−^CD204^+^CXCL9^+^) ECs was dramatically increased in CHB models (Figure 3d). HepAD38 is a widely‐used HBV‐inducible cell line in which HBV replication can be induced upon tetracycline depletion (HepAD38/tet^−^). The expression of CXCL9 in primary human liver sinusoidal endothelial cell (HLSEC) was profoundly augmented by pre‐treatment with HepAD38/tet^−^ cells (Figure S3e, Supporting Information). The recruitment of THP‐1‐drived macrophages (t‐Mφ) was enhanced by the co‐culture of HLSEC and HepAD38/tet^−^, which was reduced by CXCL9 depletion with siRNA in HLSEC (Figure 3e,f; Figure S3f, Supporting Information). As expected, the expression of *CXCR3*, the receptor of *CXCL9*, in the KCs of CHB mice and HLSECs co‐cultured with HepAD38/tet^−^ were upregulated (Figure 3g,h). Moreover, pre‐treatment with the CXCR3 antagonist AMG487 (1 µM) reduced t‐Mφ recruitment upon a gradient of CXCL9 concentrations (0, 1, 10, 100 ng mL^−1^) added to the Transwell system (Figure 3i). Multiplex imaging analysis of liver sections further demonstrated the increased aggregation of both CXCL9 and F4/80^+^ KCs with elevated CXCR3 around CV over time of CHB infection (Figure 3j; Figure S3g, Supporting Information).

Collectively, our results showed that HBV preferentially infected the peri‐central hepatocytes from an early stage of ICT, which increased CXCL9 production from neighboring ECs to recruit KCs via CXCL9‐CXCR3 axis to rupture the original peri‐portal immune zonation.

### 2.3 The Interaction of KCs with HBV^+^ Hepatocyte Induced LXR‐Mediated Lipid Accumulation in KCs

**Figure 4:**
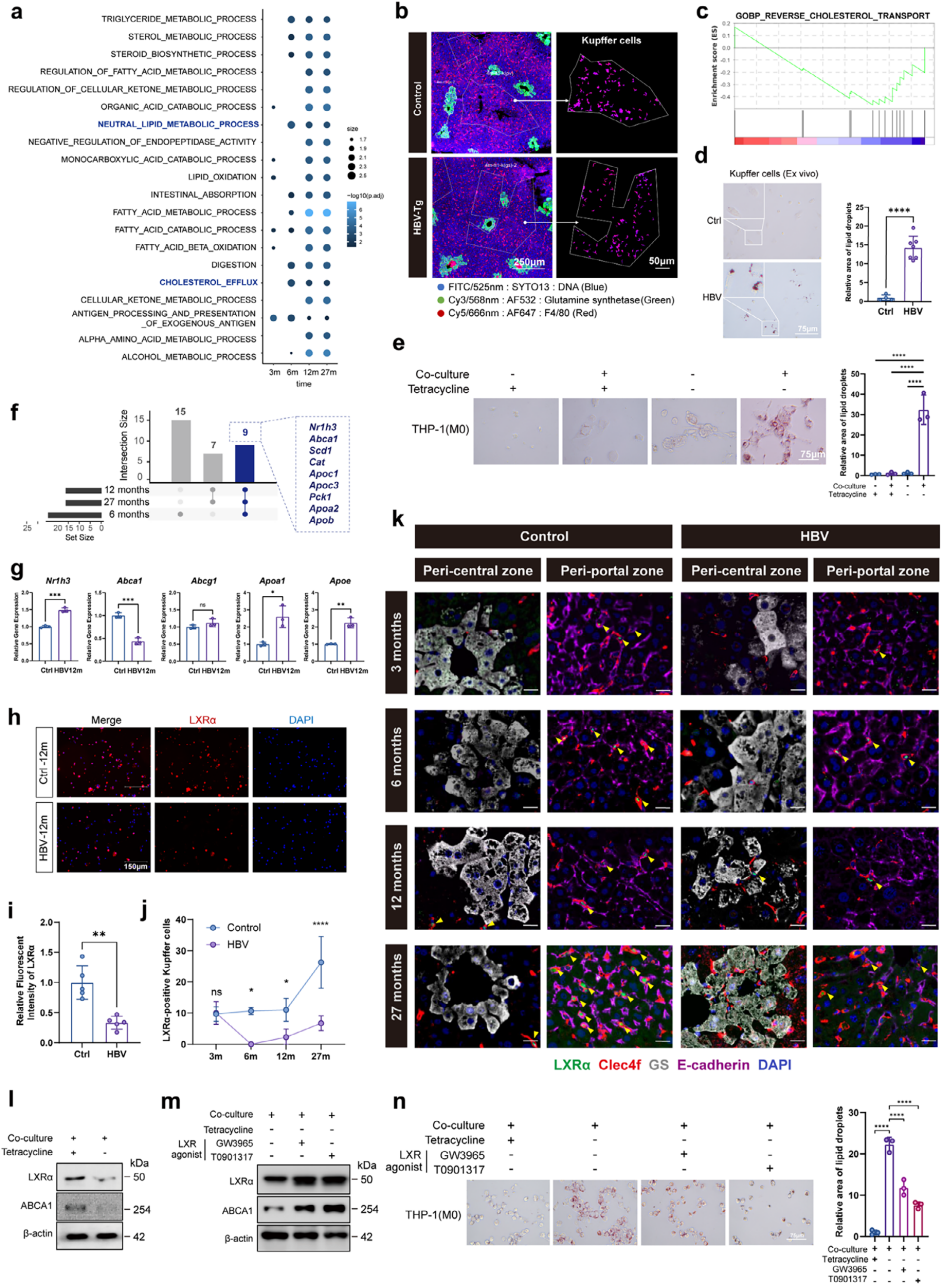
LXRα‐mediated lipid metabolism disorders in KCs upon CHB infection. a) The top 20 enriched oncology pathways by GSEA analysis of DEGs in KCs of the CHB mice versus the control mice by scRNA‐seq data. b) The representative immunofluorescent images of KC distribution and delineation of the region of interest (ROI) for DSP analysis within the FFPE sections of livers from both HBV transgenic and control mice. The antibodies for GS (green), F4/80 (red), and Syto13 (blue) were used to identify KCs with spatial information in IF assays for the subsequent transcriptome analysis of selected cells. c) GSEA analysis obtained from the DSP analysis showing dysfunctions in reverse cholesterol transport pathway in Kupffer cells of HBV transgenic mice. d) An excessive accumulation of lipid droplets in Kupffer cells dissociated from the 12‐month models. After 12 months of infection, NPCs isolated from the liver tissue were seeded in a 6‐well plate to allow macrophages to adhere, followed by Oil Red O analysis of the attached macrophages. The representative images (n≥3) were selected for the statistical analysis. e) Oil Red O analysis of THP‐1‐derived macrophages co‐cultured with HepAD38 or treated with the culture medium of HepAD38 for 48 h. Three fields of view on the representative images were selected for the statistical analysis. f) The overlapping DEGs consistently involved in the most affected lipid metabolism pathways during CHB progression. The bar plot was used to show the overlapping genes involved in the neutral lipid metabolic process and cholesterol efflux pathways in KCs among the 6‐, 12‐, and 27‐month of models. g) The mRNA level of the DEGs involved in LXRα‐related lipid processing pathways in KCs isolated from the control and mice infected with HBV for 12 months (*n* = 3). h,i) Immunofluorescent analysis (h) and quantification (i) of LXRα (red) in Kupffer cells extracted from the 12‐month AAV‐HBV and control mice. Five fields of view on the representative images were selected for the quantification of the relative fluorescent intensity of LXRα from the IF analysis of the control and CHB mice. j) The quantification of LXRα expression in KCs of the control and CHB mice. The liver tissues were isolated from the control and CHB mice of different time points, followed by IF analysis via co‐staining the anti‐LXRα and anti‐Clec4f antibodies. Three fields of view on the representative images of IF were selected from the control and CHB mice for the counting of LXRα^+^ KCs. k) Visualization of the expression of LXRα (green) in Kupffer cells (Clec4f, red) with different spatial distribution. Multiplex IF assay of the liver sections from each group at different time points were performed to show the association of LXRα in KCs with the PV (E‐cadherin, purple) or CV (GS, grey) region localization. l) Western blot analysis of LXRα and its target ABCA1 in THP‐1‐derived macrophages co‐cultured with HepAD38 (tet^‐^ or tet^+^) for 48 h. m) Activation of LXRα/ABCA1 in HepAD38‐co‐cultured Kupffer cells by LXR agonist GW3965 (10 µM) and T0901317 (10 µM). n) Oil Red O staining of lipid droplets in HepAD38‐co‐cultured THP‐1‐derived macrophages with or without LXR agonist. Three fields of view on the representative images were selected for the statistical analysis. Data are shown as mean ± SEM; *p<0.05, **p<0.01, ***p<0.001, ****p<0.0001.

As was shown, before the occurrence of HCC, the recruitment of KCs to HBV‐enriched peri‐central area benefited their interaction with HBV^+^ hepatocytes. Therefore, the functional change of KCs resulting from the crosstalk was further evaluated. By Gene Set Enrichment Analysis (GSEA, *p*‐adjust < 0.05) of KCs based on scRNA‐seq data, we found that, from 6 mpi, multiple metabolism pathways, especially lipid metabolic pathways, were enriched within the top 20 pathways, indicating KCs of the CHB mice exhibited serious lipid metabolism disorders (**Figure** **4**a; Figure S4a, Supporting Information). Transgenic mouse expressing HBV from a single integrated genome is another currently available model that is widely used in CHB study.^[^ ^31^ ^]^ By GeoMx Digital Spatial Profiler (DSP), we explored the spatial distribution and transcriptome of KCs within the region of interest in the fibrotic livers of the transgenic mice (Figure 4b; Figure S4b, Supporting Information). F4/80‐staining KCs scattered within the lobule instead of concentrating to PV region, with DEGs enriched in lipid metabolic functions by both Gene Ontology (GO) enrichment (Figure S4c, Supporting Information) and GSEA (Figure 4c). Our results showed that, along with zonation disturbance, lipid metabolism disorder (LMD) occurred in KCs in both CHB models. Accordingly, excessive accumulation of lipid drops in KCs isolated from the 12‐month AAV‐HBV mice and in t‐Mφ cocultured with HepAD38/tet^−^ were detected (Figure 4d,e).

Of note, there was no evidence that LMD in KCs could be induced by the direct effect of viral infection, as the culture medium of HepAD38/tet^−^ was insufficient to induce lipid accumulation in t‐Mφ nor the expression of HBc subunit, HBc nucleocapsids (NC), single‐strand (SS) DNA, or relaxed circular (RC) DNA in t‐Mφ (Figure 4e; Figure S4d,e, Supporting Information), which indicated a critical role of the crosstalk between HBV^+^ hepatocytes and macrophages in modulating KC lipid metabolism.

Furthermore, the genes related to the most affected lipid‐processing pathways neutral lipid metabolic process and cholesterol efflux pathways were analyzed, revealing 9 overlapping genes among the KCs derived from the mice models of 6, 12, and 27 mpi (Figure 4f). *Nr1h3* (encoding the protein of Liver X Receptor alpha (LXRα)) and *Abca1* (the downstream target gene of LXRα) were identified (Figure 4f). Actually, LXRα, in tandem with LXRβ, is the key transcriptional regulator of cholesterol, fatty acid, and phospholipid metabolism.^[^ ^32^ ^]^ The variation of *Nr1h3* was further analyzed in KCs derived from the 12‐month of CHB mice, revealing an increased mRNA level of LXRα. Additionally, the mRNA level of genes involved in LXRα‐related pathways changed with significance as well (Figure 4g). Intriguingly, in contrast to the upregulation of LXRα mRNA, the level of LXRα protein was reduced in hepatic specimens of mice infected for 3, 6, 12, and 27 months and liver‐derived KCs (Figure 4h–j; Figure S4f, Supporting Information). To further reveal the association of LXRα expression with KC zonation disturbance, we performed mIF assay in the liver tissue sections from the control and CHB mice of different time points by co‐staining of LXRα, C‐type lectin domain family 4 member F (Clec4f, KC marker), GS (CV marker), and E‐cadherin (PV marker), showing that, compared to the control mice, KCs constantly migrated to HBV‐enriched CV region with reduced LXRα expression (Figure 4k; Figure S4g, Supporting Information). The downregulation of LXRα was also demonstrated in t‐Mφ co‐cultured with HepAD38/tet^−^ cells for 48 h (Figure 4l). Moreover, activation of LXRα by LXR agonists (GW3965, T0901317) alleviated lipid accumulation in HepAD38/tet^−^‐co‐cultured t‐Mφ (Figure 4m,n).

Taken together, our results revealed that zonation disturbance‐facilitated interaction of KCs with HBV^+^ hepatocytes induced LXRα‐mediated lipid metabolism disorder in KCs.

### 2.4 The Crosstalk between LMD Macrophages and HBV^+^ Hepatocytes Caused the Formation of Hepatic Cancer Stem Cells

**Figure 5:**
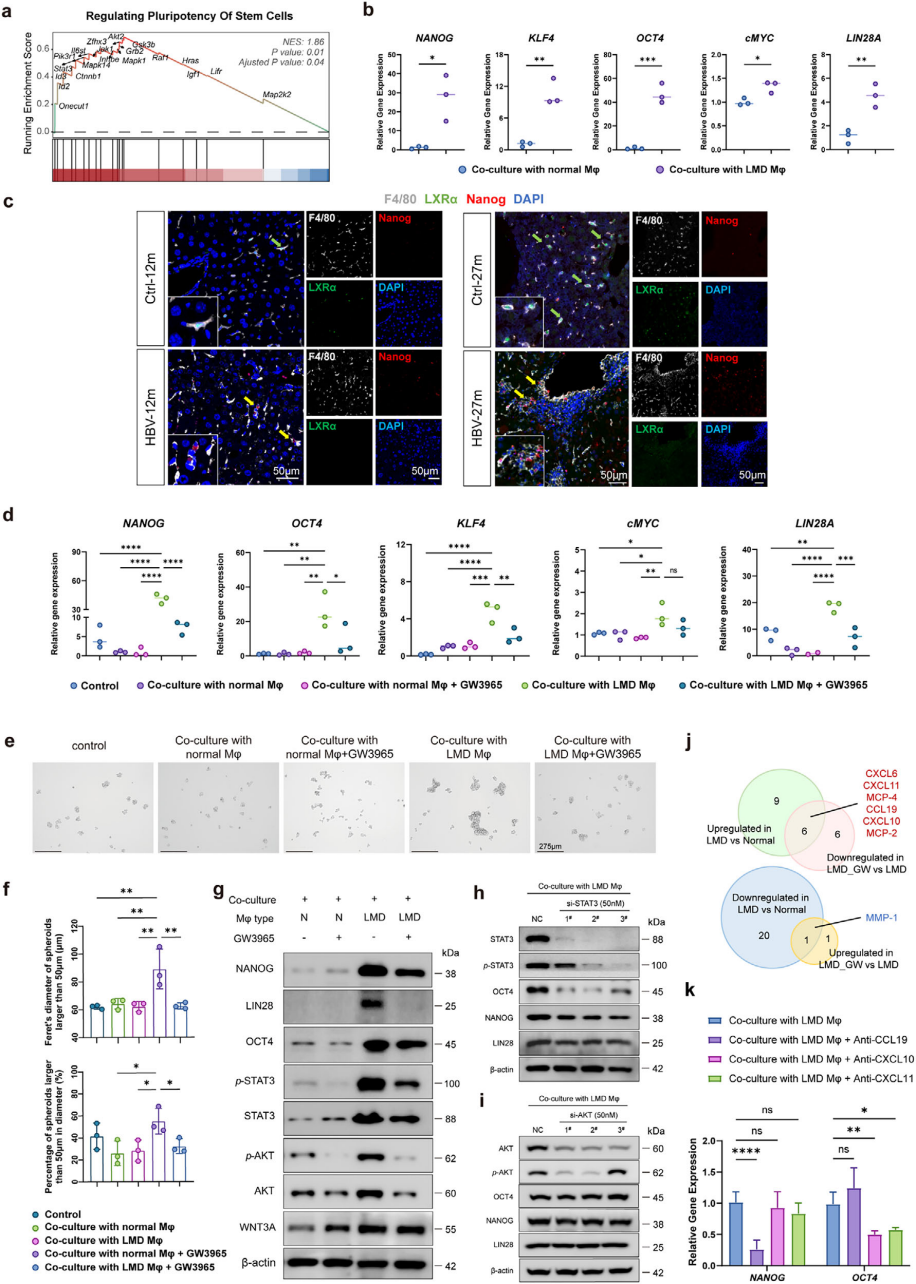
Induction of HCSC from HBV‐expressing HepAD38 cells by LMD macrophages. a) GSEA analysis showing dysregulated pathway of regulating pluripotency of stem cells in the hepatocytes of the CHB mice at 12 mpi. b) Induction of Yamanaka factors (*Nanog*, *Oct4*, *Klf4*, *c‐Myc*, *Lin28A*) in HBV‐expressing HepAD38 cells treated by macrophages with or without LMD. THP‐derived macrophages were treated with HepAD38 (tet^‐^ or tet^+^) cells for 48 h, and then co‐cultured with another well of HBV‐expressing HepAD38 in a Transwell plate for another 48 h. The experiments were conducted in triplicate (*n* = 3). c) The representative images of Nanog‐positive HCSC (red) surrounded by LXRα‐negative (low or absent of green) Kupffer cells (F4/80, grey) by mIF analysis of liver sections from the control and AAV‐HBV mice at 12 months and 27 months. d) LXR agonist GW3965 significantly reduced the increased mRNA level of pluripontency inducers in HBV‐expressing HepAD38 co‐cultured with LMD macrophages. The experiments were conducted in triplicate (*n* = 3). e) The representative bright field images of spheroids formed from HBV‐expressing HepAD38 cells treated by the normal and LMD macrophages with or without GW3965. f) The average of Feret's diameter of the spheroids and the percentage of spheroids with a diameter larger than 50 µm were analyzed. The experiments were performed in triplicate (*n* = 3). g) Western blot analysis of Yamanaka factors and the activation of STAT3, Akt, and Wnt3a signaling in HBV‐expressing HepAD38 cells treated by normal and LMD macrophages with or without GW3965. h) The induction of Nanog and Oct4 in LMD macrophage‐co‐cultured HBV^+^ HepAD38 cells was reduced by knockdown of *Stat3*. i) Knockdown of *AKT* had no effect on the expression of Nanog and Oct4 in LMD macrophage‐co‐cultured HBV^+^ HepAD38 cells. j) The Venn diagram showed the overlapping differentially expressed proteins (DEPs) exhibiting opposite variation patterns before and after GW3965 addition within the macrophage‐HepAD38 crosstalk. The supernatants of the co‐culture system involving HepAD38/tet‐ and normal or LMD macrophages with or without GW3965 were subjected to Olink analysis and 7 DEPs who had opposite changing patterns before and after GW3965 treatment were selected. k) The inhibitory effect of neutralization of CCL19, CXCL10, or CXCL11 on the expression of Nanog and Oct4 in HepAD38/tet^‐^ cells within the co‐culture system (*n* = 3–6). Data are shown as mean ± SEM; *p<0.05, **p<0.01, ***p<0.001, ****p<0.0001.

Subsequently, the effect of KCs with LMD (Mφ/LMD) on HBV‐infected hepatocytes was estimated. As mentioned above, the long‐term HBV infection induced inflammation‐to‐carcinoma transition in mice livers (Figure S1a–f, Supporting Information). More cancer hallmarks and oncogenic pathways in the hepatocytes of CHB mice were identified with the progression of the disease, and the epithelial‐mesenchymal transition (EMT) pathway was enriched in the precancerous 12‐month of mice models (Figure S5a, Supporting Information). EMT has been related to the generation and maintenance of cancer stem cells. Actually, in the 12‐month of CHB mice, the regulation of pluripotency of stem cells was enhanced in hepatocytes (**Figure** **5**a). Hepatic cancer stem cells (HCSC) are a small population of stem‐like hepatocarcinoma cells that could initiate HCC.^[^ ^33^ ^]^ Since the production of CSC by Yamanaka factors, a group of transcription factors that play pivotal roles in generating induced pluripotent stem cells, is well accepted,^[^ ^34^ ^]^ we examined the expression of Yamanaka factors in HepAD38/tet^−^ upon t‐Mφ treatment and found that Mφ/LMD induced a dramatic increase in the mRNA level of *Nanog*, *Oct4*, *Klf4*, *cMyc* and *Lin28A* (Figure 5b). Nanog, functioning in the initiation and self‐renewal of HCSC, has been suggested as a biomarker for liver CSC.^[^ ^35^ ^]^ In CHB livers of different time points, Nanog^+^ HCSCs were detected to be embraced by LXRα‐deficient F4/80^+^ KCs, indicating that LMD KCs participated in pro‐CSC niche formation (Figure 5c).

Moreover, in response to GW3965, the expression of Yamanaka factors in HepAD38/tet^−^ co‐cultured with Mφ/LMD declined to approximately the control level (Figure 5d). Notably, GW3965 had no direct inhibitory effect on Yamanaka factors in HepAD38/tet^−^ cells pre‐treated with Mφ/LMD, which confirmed that the reversal effect of GW3965 on CSC inducers was attributed to LXRα activation in Mφ/LMD within the co‐culture system (Figure S5b, Supporting Information). Additionally, GW3965 also affected the spheroid formation capacity of HepAD38/tet^−^ endowed by Mφ/LMD, evidenced by a reduction of the size and number of the spheroid (Figure 5e,f). Furthermore, we employed flow cytometry analysis to further validate the stemness of the cells. Briefly, HepAD38 cells (tet^−^), pre‐cocultured with THP‐1 macrophages with or without LMD were collected and subjected to flow cytometry analysis of the well‐established liver CSC markers^[^ ^36^, ^37^ ^]^ (CD133, CD90). As shown in the revised Figure S5c (Supporting Information), percentages of CD133^+^ and CD90^+^ cell population were significantly induced in HepAD38 cells cocultured with LMD THP‐1 macrophages.

Furthermore, the activation of Stat3, Wnt, and phosphoinositide 3‐kinase (PI3K)/Akt signaling pathways, whose roles in modulating pluripotent factors and CSC formation have been well established,^[^ ^38^, ^39^, ^40^ ^]^ were identified in the enriched pathway of the regulation of pluripotency of stem cell (Figure 5a). In addition to the induction of Nanog, Lin28A, and Oct4, Mφ/LMD dramatically up‐regulated the phosphorylation of Stat3 and Akt in HepAD38/tet^−^, which could also be suppressed by GW3965 (Figure 5g). However, the elevation of Nanog and Oct4 in HepAD38/tet^−^ cells induced by Mφ/LMD was repressed by *Stat3* knockdown rather than *Akt* inhibition, with Oct4 exhibiting higher sensitivity to Stat3 signaling, which indicated that Mφ/LMD‐induced pluripotent factors upregulation was partially mediated by Stat3 signaling (Figure 5h,i). Besides its pro‐CSC capacity, Mφ/LMD also participated in fibrosis formation, as the mRNA level of *transforming growth factor beta 1 (TGF‐β1)* and the *MMP‐2* were increased in HepAD38/tet^−^ cells co‐cultured with Mφ/LMD and reduced significantly upon GW3965 (Figure S5d,e, Supporting Information).

Since there was no physical contact between macrophages and hepatocytes in the pro‐CSC co‐culture system, we performed Olink inflammation panel analysis allowing simultaneous estimation of 92 analytes to profile potential inflammatory biomarkers within the supernatant from the crosstalk between HepAD38/tet^−^ and macrophages with and without GW3965 (Figure S5f, Supporting Information). Compared to the co‐culture with normal macrophage (co‐normal), 36 differentially expressed proteins (DEPs) were identified in Mφ/LMD co‐culture system (co‐LMD), of which 15 DEPs were upregulated and 21 downregulated (Figure S5g, Supporting Information). The addition of GW3965 (co‐LMD/GW3965) induced 2 DEPs to be upregulated and 12 downregulated within the co‐LMD system (Figure S5h, Supporting Information). Six overlapping DEPs (C‐C motif chemokine ligand (CCL) 8, CCL13, CCL19, C‐X‐C motif ligand (CXCL) 6, CXCL10, and CXCL11) whose concentrations were profoundly higher in co‐LMD than in co‐normal condition and sharply reduced upon GW3965 treatment were identified to play potential roles in mediating Mφ/LMD‐induced CSC formation (Figure 5j; Figure S5j, Supporting Information). CCL19, CXCL10, and CXCL11, with higher degree scores via protein‐protein interaction and correlation analysis, possessed the greatest concentration varying patterns among different conditions (Figure S5i–k, Supporting Information). Furthermore, CCL19 neutralization reduced the elevation of Nanog induced by Mφ/LMD in HepAD38/tet^−^, while blockade of CXCL10 and CXCL11 preferentially inhibited Oct4 expression (Figure 5k). Conclusively, the pro‐CSC effect of Mφ/LMD on HBV^+^ hepatocytes was suggested to rely on the chemokine network within the co‐culture system, with CCL19, CXCL10, and CXCL11 functioning as core mediators to modulate the pluripotency genes.

Overall, our results indicated that LMD macrophages induced fibrosis and CSCs formation from HBV^+^ hepatocytes through cell‐cell crosstalk, which was mediated by the chemokine network‐induced activation of Stat3 signaling.

### 2.5 LXRα Reduction in KCs was Regulated by MiR‐155 and ASGR1 and Intimately Associated with HCC in HBV^+^ Patients

**Figure 6:**
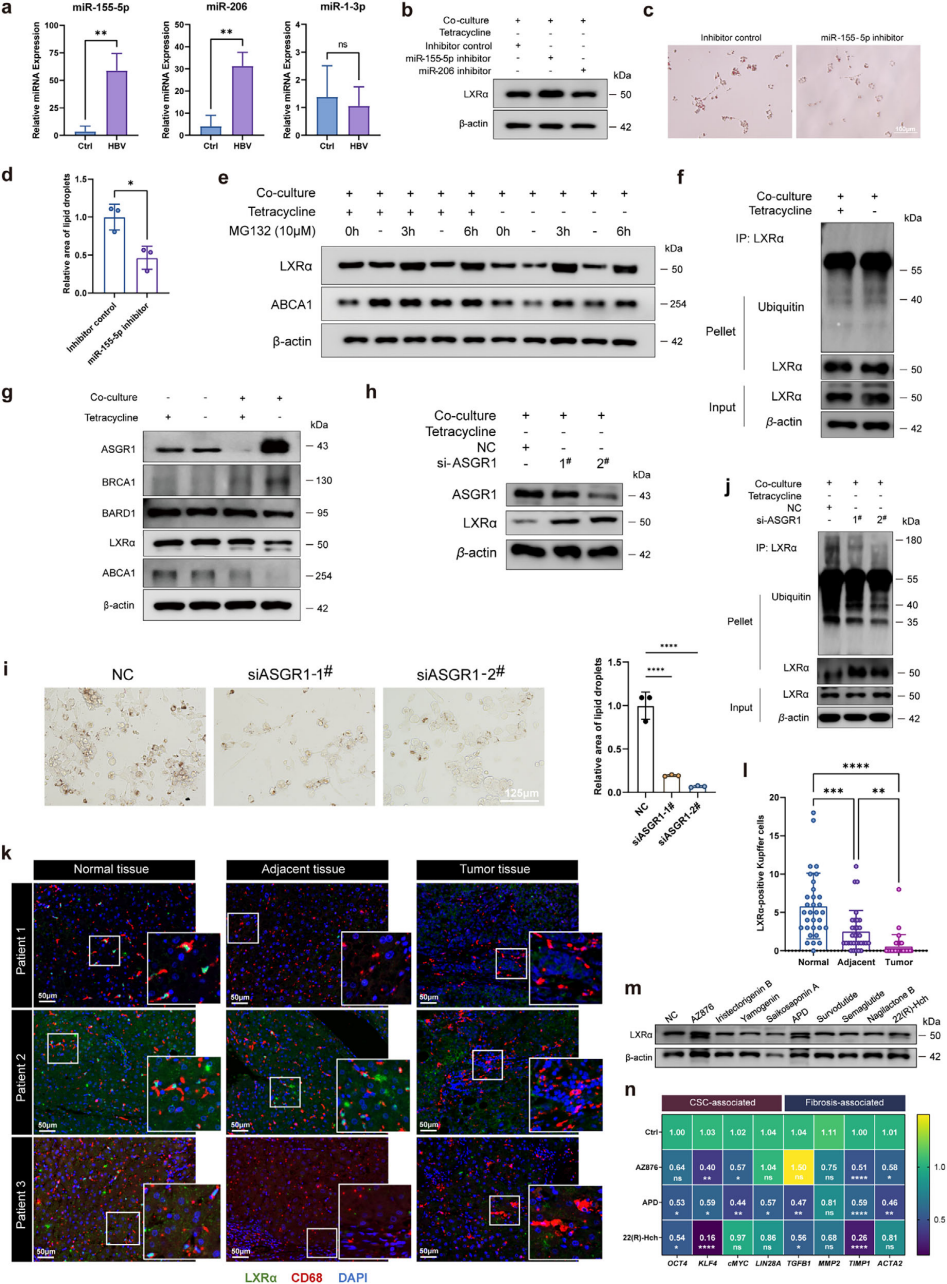
MiR‐155‐mediated post‐transcriptional regulation and ASGR1‐mediated protein degradation cooperatively contributed to reduce the protein level of LXRα in KCs. a) The relative expression of miR‐155‐5p, miR‐206, and miR‐1‐3p in Kupffer cells isolated from the control and the 12‐month of CHB mice (*n* = 3 or 4). b) The effect of miR‐155 inhibitor or miR‐206 inhibitor on the expression of LXRα in HepAD38/tet^‐^‐cocultured macrophages. THP‐derived macrophages were pre‐treated with miR‐155‐5p inhibitor or miR‐206 inhibitor and then co‐cultured with tetracycline‐off HepAD38, followed by Western blot analysis of LXRα protein in the KCs. c,d) MiR‐155 inhibitor decreased lipid accumulation in KCs co‐cultured with HBV‐expressing HepAD38. e) Western blot analysis of LXRα and ABCA1 in HepAD38‐treated macrophages with or without MG132 (10 µM) treatment for indicated time. f) THP‐1‐derived macrophages were co‐cultured with HepAD38 (tet^‐^ or tet^+^) for 48 h. Then, the cells were incubated with 10 µM MG132 for 6 h and lysed for immunoprecipitation with anti‐LXRα antibody coupled protein A/G to pull down LXRα. The Input and pellet fractions were subjected to Western blot analysis and immunoblotted with anti‐LXRα, anti‐ubiquitin, and anti‐*β*‐actin antibodies. g) Western blot analysis of genes involved in ASGR1‐associated LXRα degradation pathways in THP‐derived macrophages with or without HepAD38 coculture. Cell lysate of THP‐derived macrophages cocultured with HepAD38 (tet^‐^ or tet^+^) or treated with the culture medium of HepAD38 (tet^‐^ or tet^+^) was subjected to Western blotting to examine ASGR1‐mediated LXRα degradation signaling. h,i) Knockdown of *ASGR1* by siRNA significantly reversed HepAD38/tet^‐^‐induced LXRα reduction and lipid accumulation in THP‐derived macrophages. THP‐derived macrophages pre‐treated with NC or ASGR1 siRNAs for 24 h were co‐cultured with HBV‐expressing HepAD38 cells and then subjected to Western blot analysis of LXRα expression h) and Oil O Red staining of lipid droplets i) in macrophages. j) THP‐1‐derived macrophages were transfected by siASGR1 for 24 h, followed by co‐cultured with HepAD38 (tet^‐^) for 48 h. Then, the cells were incubated with 10 µM MG132 for 6 h and lysed for immunoprecipitation with anti‐LXRα antibody coupled protein A/G to pull down LXRα. The Input and pellet fractions were subjected to Western blot analysis and immunoblotted with anti‐LXRα, anti‐ubiquitin, and anti‐*β*‐actin antibodies. k,l) IF analysis (k) and quantification (l) of LXRα (green) expression in KCs (CD68, red) residing in the normal, tumor adjacent, and tumor tissue of the liver microarray of HBV^+^ patients with HCC. The expression of LXRα in the KCs within the tumor tissue was the lowest compared to that of the normal and adjacent tissues (k). The LXRα^+^ KCs in the normal, adjacent, and tumor tissues of the patient liver microarray was statistically analyzed (l). m) The effect of different agents on the expression of LXRα in THP‐1‐derived macrophages. THP‐1‐derived macrophages were treated with AZ876, Iristectorigenin B, Yamogenin, Saikosaponin A, APD, Survodutide, Semaglutide, Nagilactone B, or 22(R)‐Hch for 48 h, respectively, followed by Western blot analysis of LXRα. n) The effects of the selected agents on the expression of fibrosis‐ and CSC‐associated marker genes in HepAD38 cells induced by the co‐culture with LMD macrophages. THP‐1‐derived LMD macrophages were pre‐treated with AZ876, APD, or 22(R)‐Hch for 48 h before co‐cultured with HepAD38/tet^‐^ for 48 h, followed by real‐time PCR analysis of the fibrosis marker genes (*TGF‐ß*, *MMP‐2*, *TIMP1*, *ACTA2*) and Yamanaka factors. The experiments were conducted in triplicate (*n* = 3). Data are shown as mean ± SEM; *p<0.05, **p<0.01, ***p<0.001, ****p<0.0001.

Finally, we came to elucidate the potential molecular mechanisms dominating the differential expression pattern of LXRα mRNA and protein in KCs under CHB infection (Figure 4). Post‐transcriptional mechanisms and protein degradation are widely accepted to be two major avenues to shape protein levels independently of mRNA abundance.^[^ ^41^ ^]^ On one hand, as microRNAs (miRNAs) regulate gene expression at post‐transcriptional level, we analyzed the known LXRα‐targeting miRNAs (miR‐155, miR‐206, and miR‐1) in KCs derived from the 12‐month mice. Our results showed that miR‐155 and miR‐206 were upregulated in HBV‐treated group (**Figure** **6**a), whereas only suppression of miR‐155 increased LXRα protein and reduced lipid droplet accumulation in t‐Mφ co‐cultured with HepAD38/tet^−^ cells (Figure 6b–d).

On the other hand, the potential role of protein degradation in CHB‐associated LXRα protein reduction was also assessed. The addition of proteasome inhibitor MG132 reversed LXRα reduction in macrophages treated with HepAD38/tet^−^, indicating that HBV^+^ HepAD38 cells induced LXRα degradation in a proteasome‐dependent manner (Figure 6e). The ubiquitination of LXRα in macrophages was induced by HepAD38/tet^−^ treatment (Figure 6f). More recently, asialoglycoprotein receptor 1 (ASGR1) was reported to regulate lipid metabolism by degrading LXRα, and ASGR1 deficiency decreased lipid contents in serum and liver by stabilizing LXRα through decreasing its ubiquitin ligase breast cancer gene (BRCA) 1/ BRCA1 Associated RING Domain (BARD) 1^42^. Therefore, we analyzed ASGR1‐related LXRα degradation pathway in HepAD38/tet^−^‐cocultured t‐Mφ to show an extremely high level of ASGR1, accompanied by BRCA1 elevation and LXRα‐ABCA1 decline (Figure 6g). Notably, the culture medium of HepAD38/tet^−^ was less efficient in affecting the ASGR1‐LXRα signaling of macrophages, which again demonstrated that the interplay between macrophages and infected hepatocytes contributed to LXRα reduction and the related lipid metabolism disorders in macrophages during CHB (Figure 6g). Moreover, ASGR1 deficiency by siRNA restored the protein level of LXRα by reducing its ubiquitination degradation pathway, rather than its transcriptional level (Figure 6h–j).

As we revealed the role of LXRα in promoting inflammation‐to‐carcinoma transition in CHB mice models, we further evaluated if the dysregulation of LXRα in KCs was associated with HCC in HBV^+^ patients. Using the liver tissue microarray of HBV^+^ patients with HCC (**Table** **1**; Figure S6a, Supporting Information), we found that the expression of LXRα was almost depleted in infiltrated KCs within the tumor tissue compared to that of the adjacent tissue, while KCs of the normal tissue exhibited the highest expression of LXRα, which indicated that the level of LXRα in KCs was closely related to HCC (Figure 6k,l). To better illustrate the potential therapeutic role of LXRα in CHB‐associated liver diseases, the agents capable of stimulating LXRα were examined for their capability of activating LXRα in KCs, and AZ876, Acetyl podocarpic acid anhydride (APD), and 22(R)‐Hydroxycholesterol (22(R)‐Hch) were potent in increasing LXRα expression in THP‐1‐derived macrophages (Figure 6m). Moreover, AZ876, APD, and 22(R)‐Hch were proved to reduce Mφ/LMD‐increased marker genes for CSC and fibrosis (Figure 6n).

**Table 1:** Basic information, tumor characteristics and pathologic data.

Parameter	No. of patients [*n* = 32]
Age	
≤ 50	15 (46.8%)
>50	17 (53.1%)
Gender	
Male	25 (78.1%)
Female	7 (21.9%)
Disease	
HCC	29 (90.6%)
ICC	3 (9.4%)
Grade	
GII	27 (84.4%)
GIII	2 (6.2%)
/	3 (9.4%)
T stage	
T2	29 (90.6%)
T3	2 (6.2%)
/	1 (3.1%)
Number of tumors	
Single	28 (87.5%)
Multiple	4 (12.5%)
Diameter of maximum tumor	
≤ 5 cm	24 (75.0%)
>5 cm	8 (25.0%)
HBV infection	32 (100.0%)
Negative	0 (0%)
Positive	100 (100%)
Natural history of hepatitis B	
immune‐tolerance	27 (84.4%)
quiescent	1 (3.1%)
/	4 (12.5%)
Pathologic diagnosis	
G1S3	2 (6.2%)
G2S3	2 (6.2%)
/	28 (87.5%)
Laennec staging	
F4A	8 (25.0%)
F4B	2 (6.2%)
F4C	15 (46.9%)
/	7 (21.9%)
HBsAg	
−	3 (9.4%)
+	19 (59.4%)
++	7 (21.9%)
+++	2 (6.2%)
±	1 (3.1%)
HBcAg	
−	30 (93.8%)
+	2 (6.2%)
	
GPC‐3	
−	2 (6.2%)
+	18 (56.2%)
++	12 (37.5%)
CD10	
−	17 (53.1%)
+	15 (46.9%)
CD8	
+	32 (100.0%)
AFP	
−	32 (100.0%)
CK7	
−	28 (87.5%)
+	3 (9.4%)
++	1 (3.1%)
CD19	
−	28 (87.5%)
+	2 (6.2%)
++	2 (6.2%)
CK20	
−	26 (81.2%)
+	5 (15.6%)
++	1 (3.1%)
CD34	
−	1 (3.1%)
+	31 (96.9%)
Ki67	
10%+	3 (9.4%)
20%+	7 (21.9%)
30%+	5 (15.6%)
40%+	6 (18.8%)
50%+	3 (9.4%)
60%+	3 (9.4%)
70%+	3 (9.4%)
75%+	1 (3.1%)
80%+	1 (3.1%)
CD117	
−	32 (100.0%)
CEA	
−	27 (84.4%)
+	5 (15.6%)
EMA	
−	25 (78.1%)
+	6 (18.8%)
++	1 (3.1%)
Vimentin	
−	31 (96.9%)
+	1 (3.1%)
VEGFR	
−	12 (37.5%)
+	20 (62.5%)
CgA	
−	32 (100.0%)
SyN	
−	32 (100.0%)
CDX‐2	
−	26 (81.2%)
±	6 (18.8%)
HSP70	
−	4 (12.5%)
+	19 (59.4%)
0.5+	9 (28.1%)

Collectively, the decrease of LXRα protein in KCs was caused by ASGR1‐mediated degradation and miR‐155‐involved post‐transcriptional regulation during CHB progression. LXRα‐deficiency in KCs was intimately associated with HCC in HBV‐positive patients. Importantly, the strategy of modulating LXRα expression is probably efficacious for the intervention of CHB malignancy.

## 3 Discussion

CHB infection, causing persistent liver inflammation, could lead to cirrhosis and ultimately HCC, yet the underlying principles of which remain largely uncovered. KCs, as the most abundant liver‐resident immune cells, exhibit a central effect on hepatic homeostasis and each pathological stage that would also occur during CHB progression. Here, for the first time, we generated the kinetic landscape of KC transcriptomics across time and space during CHB progression, through which a novel “trilogy” mechanism of KCs in governing CHB‐related pathogenesis was revealed: 1) the peri‐portal zonation of KC was disturbed to facilitate the crosstalk between hepatocytes and KCs within HBV‐enriched peri‐central area; 2) the interplay between HBV^+^ hepatocytes and macrophages caused lipid metabolism disorders in KCs; 3) in turn, KCs with LMD induced HCSC formation from HBV‐expressing hepatocytes.

The AAV‐HBV mouse model, generated by delivering HBV genome into mouse hepatocytes via hepatotropic adeno‐associated virus, achieves prolonged high‐level of HBV viremia and antigenemia, and presents fibrosis and liver tumorigenesis, which makes it an appropriate animal model for CHB study and therapeutic evaluation.^[^ ^27^, ^43^, ^44^ ^]^ Consistently, the AAV8‐HBV mouse model we used here successfully progressed from inflammation to fibrosis after six months of infection and finally developed into severe fibrosis and HCC, paving the way for solid data collection.

Immune zonation in heathy liver was just reported, with KCs preferentially aggregating around the portal inlets,^[^ ^20^, ^21^, ^45^ ^]^ which was also observed in our data (Figure 2g). The peri‐portal localization of KCs is considered to benefit their role in capture of pathogens entering through PV, protecting the downstream hepatocytes from being infected.^[^ ^46^, ^47^ ^]^ While, in CHB mice, we found that HBV‐infected hepatocytes were more readily to be present within the peri‐central outlet (Figure 2a–c), resulting from the hypoxia microenvironment around CV that favors HBV replication^[^ ^48^ ^]^ and an inefficient eradication of the virus by KCs at the peri‐portal barrier. The latter hypothesis was supported by our data, as more KCs of CHB mice were peri‐centrally recruited (Figure 2e,f; Figure S2c, Supporting Information). Hepatic macrophage zoning is being recognized to relate to liver homeostasis and pathologies. In non‐alcoholic fatty liver disease (NAFLD), a population of Gpnmb^+^Spp1^+^ lipid‐associated macrophage was revealed to aggregate within the peri‐central region where steatosis was detected.^[^ ^21^ ^]^ Moreover, another progression‐associated macrophage subtype defined as IBA1^+^CD163^low^CD16^low^ was found to locate around the ductular cells within nonparenchymal areas.^[^ ^22^ ^]^ However, no data has been published regarding the role of liver terrain in the progression of CHB. Therefore, in this study, we focused on the molecular motivation and consequence of KC zonation disturbance during CHB infection.

By analyzing the spatial expression pattern of macrophage‐recruiting chemokines with their cell origins through ST data and mIF assays, we found that only CXCL9, derived from LSECs, was significantly increased within HBV‐infected peri‐central area, in line with which, primary HLSECs pre‐treated with HepAD38/tet^−^ also enhance the migration of macrophages through CXCL9 (Figure 3b–f). Additionally, CXCR3 was increased in KCs from CHB models, indicating a peri‐central activation of CXCL9‐CXCR3 axis. Recently, the pathway of Notch^[^ ^49^ ^]^ and MyD88^[^ ^20^ ^]^ in LSECs and ALK1 signaling in Kupffer cells^[^ ^21^, ^23^ ^]^ have been proposed to contribute to the aggregation of KCs around PV in normal conditions, with LESC producing a CXCL9 gradient along the porto‐central axis. Combining the published data and the spatial gradient of CXCL9 contrary between the control and CHB mice, we proposed that CXCL9‐CXCR3 axis is the driving force for KC distribution within the liver.

Of note, macrophages residing in different hepatic zonation perform varied functions.^[^ ^50^ ^]^ We found that, upon CHB‐induced zoning disturbance, multiple metabolic processes, especially lipid metabolism pathways as neutral lipid metabolic process and cholesterol efflux, were dysregulated in KCs (Figure 4a). In addition, lipid metabolism dysfunction was also demonstrated in KCs of HBV transgenic mice with liver fibrosis (Figure 4b,c), indicating the occurrence of LMD in KCs was not unique to a given CHB model. Furthermore, *Nr1h3*, the coding gene for LXRα which plays critical roles in cholesterol reverse transport, was dysregulated in the KCs of CHB mice. Notably, the protein level of LXRα in KCs infiltrated into the peri‐central area was persistently reduced independently of its mRNA expression (Figure 4h–k) and LXR agonists dramatically repressed lipid accumulation in Mφ/LMD (Figure 4l–n), highlighting the central role of LXRα in CHB‐associated lipid metabolism disorder. In other cell types, microRNAs and ASGR1 have been reported to regulate LXRα abundance after mRNA is made.^[^ ^42^, ^51^, ^52^, ^53^ ^]^ We found that miR‐155‐mediated post‐transcriptional regulation and ASGR1‐mediated protein degradation collaboratively modulated LXRα expression during CHB progression. However, unlike the potency of ASGR1 depression in decreasing lipid levels by stabilizing LXRα to promote cholesterol excretion in hepatocytes, in our study, ASGR1 suppression played only a partial role in reversing LMD, indicating a more complicated regulatory network in the lipid metabolism of KCs.

Lipid metabolism has been indicated in shaping KC functions,^[^ ^54^, ^55^, ^56^, ^57^ ^]^ with LXRα playing critical roles. It was previously highlighted that LXRα was required to maintain KC‐specific identity,^[^ ^58^, ^59^ ^]^ and down‐regulation of LXRα could induce KC identity loss and contribute to liver fibrosis.^[^ ^15^, ^60^ ^]^ In our study, Mφ/LMD was also revealed to be capable of increasing the expression of fibrosis biomarkers *TGF‐β1* and *MMP‐2*, which was obviously reversed by LXRα activation (Figure S5d,e, Supporting Information). Furthermore, precancerous lesions and more oncogenic pathways were detected in the 12‐month CHB model (Figure 5a; Figure S5a, Supporting Information), and HCSCs were detected to be embraced by a couple of LXRα‐deficient KCs within the liver (Figure 5c). The effect of Yamanaka factors on HCC and liver CSC development and maintenance has been well‐accepted.^[^ ^34^, ^61^ ^]^ We found that, in HepAD38/tet‐ cells, the spheroid formation capacity and the expression of Yamanaka factors were enhanced by Mφ/LMD via Stat3 and AKT signaling, which could be efficiently inhibited by GW3965. Furthermore, GW3965 could efficiently reverse Mφ/LMD‐induced “stemness” formation in HepAD38/tet‐ cells. Our results revealed a critical role of LXRα‐mediated lipid metabolism of KCs in CHB‐associated CSC formation, which further support the notion that HBV‐induced hepatic chronic inflammation functions as a key regulator in HCC initiation.^[^ ^62^, ^63^ ^]^

In addition, cytokine/chemokine‐mediated cellular interplay is intricately correlated with the progression of multiple cancers.^[^ ^64^, ^65^, ^66^ ^]^ A series of chemokines whose expression was robustly elevated within the crosstalk between HBV‐expressing hepatocytes and Mφ/LMD yet reversed following GW3965 treatment, were uncovered. CXCL6, in tandem with cardiotrophin‐like cytokine factor 1 (CLCF1), has been reported to function in the crosstalk among cancer‐associated fibroblasts, tumor cells, and tumor‐associated neutrophils to aid in cancer stem cell formation and poor prognosis in HCC.^[^ ^64^ ^]^ The secretion of CXCL10 from HSCs has been shown to promote M1 polarization of macrophage through CXCR3, leading to liver fibrosis progression.^[^ ^67^ ^]^ In our pro‐CSC network, selective blockade of CXCL6 did not interfere with CSC generation from HepAD38, while CCL19, CXCL10, or CXCL11 were revealed to be potent in regulating the induction of pluripotency factors (Figure 5k).

Additionally, the crosstalk between KCs and other cells within the liver has been suggested by other groups. KC niche composing of stellate cells, hepatocytes, and endothelial cells imprinted KC identity by inducing the expression of liver‐associated transcription factors inhibitor of DNA 3 (ID3) and LXRα in recruited circulating monocytes.^[^ ^68^, ^69^ ^]^ Moreover, in liver pathologies as nonalcoholic steatohepatitis (NASH), fibrosis, and acute liver injury, the crosstalk among KC and surrounding cells was indicated to function in disease progression.^[^ ^70^, ^71^, ^72^ ^]^ In our study, the zonation alteration of KC offered the spatial structure basis for the communication of KC with HBV‐positive hepatocytes within the peri‐central area. Moreover, only co‐culture with HepAD38/tet^−^ cells, rather than with HepAD38/tet^−^ culture medium, induced lipid metabolism dysfunction in KCs (Figure 4e). At the same time, the inhibitory role of GW3965 in the induction of Yamanaka factors in HepAD38/tet^−^ cells was attributed to its activation of LXRα in Mφ/LMD cells, instead of its direct effect on HepAD38 cells (Figure 5d; Figure S5b, Supporting Information). It has been recently reported that lipid‐laden macrophages directly transfer myelin‐derived lipids to tumor cells via LXR/Abca‐1 to fuel the high metabolic demand of mesenchymal glioblastoma, indicating the critical role of LXR‐mediated lipid metabolism in the crosstalk between macrophages tumor cells.^[^ ^73^ ^]^ Our study revealed that the immune zonation disturbance‐facilitated cell‐cell interaction is critical to shape the phenotype and function of both KC and hepatocyte upon prolonged HBV infection.

Finally, by analyzing the expression of LXRα of KCs in the tissue microarray of HCC patients with HBV infection, we found that, compared to the normal and peri‐tumor tissues, LXRα was significantly reduced in the tumor tissues, which indicated a close association between LXRα‐deficient KCs and HCC. Regarding LXRα was persistently reduced from an early stage of CHB‐associated liver diseases and LXRα‐mediated LMD in KCs was potent in inducing CSC formation, LXRα in KCs represented as a promising target for the early intervention of the disease progression. And thus, several reported LXRα agonists were examined in our study, showing a beneficial effect on maintaining LXRα expression in KCs co‐cultured with HBV^+^ hepatocytes and preventing the induction of fibrosis and CSC generation (Figure 6m,n). However, extensive studies, especially in vivo experiments, are required to validate the potential of LXRα‐targeting strategies against CHB‐induced hepatopathologies. Given the critical role of LXR in lipid metabolism, LXR agonists have been implied in the treatment of lipid metabolism‐associated diseases. Therefore, the side effects of LXR agonists are being focused.^[^ ^74^, ^75^ ^]^ It has been reported that pan‐LXR agonists elicit significant TG elevation. Moreover, hepatic steatosis, hypertriglyceridemia, and elevated LDL‐cholesterol are also revealed to be induced by LXR agonists in primates. To address this challenge, several selective LXR agonists (e.g., LXR‐623, Wyeth Pharmaceuticals – now Pfizer; BMS‐779788, XL‐652; Exelixis and Bristol‐Myers Squibb; BMS‐852927, XL‐041; Exelixis and Bristol‐Myers Squibb) have been developed and tested in clinical trials. However, the clinical trial of LXR‐623 was halted despite its efficacy in upregulating LXR target genes in peripheral blood cells, as dose‐limiting central nervous system (CNS) and psychiatric adverse events emerged at the two highest doses.^[^ ^76^ ^]^ These data imply that the side effects of LXR agonists are a major obstacle of its clinical use. In this study, we found that upregulation of LXRα was a promising therapeutic strategy for early intervention of inflammation‐to‐tumor transition induced by CHB infection. It is therefore crucial to understand the potential side effects of LXR agonists to facilitate their translation from bench to bedside.

In conclusion, the dynamic landscape of hepatic parenchymal and non‐parenchymal cells across time and space during CHB‐induced ICT was mapped for the first time in this study. Our findings shed new lights on the vital role of KC zonation, as well as lipid metabolism, in CHB‐related liver diseases, providing potential therapeutic targets for innovative interventions against the progression of CHB.

## 4 Experimental Section

### Cell Culture

THP‐1 cells were maintained in RPMI‐1640 medium (Corning) supplemented with 10% fetal bovine serum, 100 U mL^−1^ penicillin‐streptomycin and 0.05 mM β‐Mercaptoethanol (β‐Me; Sigma). THP‐1 cells were differentiated into macrophages by incubation in the presence of 100 nM phorbol 12‐myristate 13‐acetate (PMA; Sigma) for 24 h, followed by incubation in fresh medium without PMA. HepAD38 cells were maintained in Dulbecco's modified Eagle's medium (DMEM; Corning) supplemented with 10% fetal bovine serum, 50 ug mL^−1^ of penicillin‐streptomycin, 400 µg mL^−1^ of G418 and 1 µg mL^−1^ of tetracycline. Human Liver Sinusoidal Endothelial Cells (HLSECs) were obtained from Meisen Chinese Tissue Culture Collections (MeisenCTCC, Zhejiang, China), and cultured in endothelial cell growth medium supplemented with 10% fetal bovine serum. To study the mechanisms of macrophages exposed to HBV infection, THP‐1 were pre‐plated into a 6‐well plate and treated immediately with 100 nM PMA for 24 h. Once settled THP‐1 macrophages, the Transwell inserts containing HepAD38 cells were placed into a 6 well plate to set up the co‐culture system for 2 days. As for neutralization of chemokines in vitro, antibodies neutralizing CCL19 (2 µg mL^−1^, Invitrogen #PA5‐46940), CXCL10 (0.5 µg mL^−1^, Invitrogen, #MA5‐23819), or CXCL11 (0.5 µg mL^−1^, Invitrogen, #MA5‐23761) were pretreated in the t‐Mφ‐HepAD38 co‐culture system. All cells were cultured at 37 °C in an atmosphere of 5% CO_2_ level.

### Mice

Normal C57BL/6J (male; 6–8 weeks of age) mice were provided by Beijing Vital River Laboratory Animal Technology Co., Ltd (License No. SCXK‐(jing) 2016‐0006) and bred in the Animal Facility of the Department of the Academy of Military Medical Sciences. Mice ranging from 3–27 mpi were used throughout the study. Animal care and procedures were in accordance with the Animal Welfare guidelines (Approval No. IACUC‐DWZX‐2020‐667).

### Human Liver Tissue Microarray

Human liver tissues were obtained from the Shanghai Zhuoli Boitech Company under ethics approval (No. SHLLS‐BA‐22101102). All samples were anonymized prior to analysis by removing patient identifiers and using coded labels as Patient 1–32 in Table 1. Consent was waived by the ethics committee as samples were retrospectively collected from diagnostic archives. The experiments complied with the ethical principles of the Declaration of Helsinki.

Briefly, the human liver tissue microarrays were produced using multiple donor paraffin blocks by first creating a new, empty recipient paraffin block. Target regions were carefully identified on Hematoxylin and Eosin (H&E)‐stained slides corresponding to each donor block. A specialized tissue arrayed equipped with a hollow needle, precisely aligned with the marked area on a donor block, was then used to extract a thin core of tissue. Simultaneously, an identical core was removed from the recipient block to create an empty hole. The donor tissue core was immediately inserted into this precisely arranged position within the recipient block. This process was systematically repeated, transferring representative cores from specific areas of each unique donor block into pre‐defined, organized positions on the single recipient block.

### Construction and Characterization of HBV Infection in AAV‐HBV Mice

AAV‐HBV vector that packages a 1.3‐kb genome of the D genotype HBV ayw in AAV serotype 8 (AAV8) capsids was provided by Guangzhou PackGene Biotech Institute (Guangzhou, China). Wide‐type C57BL/6J mice were injected with 1.0E11GE AAV‐HBV in 200 µL of phosphate‐buffered saline (PBS) through tail vein. HBV‐infected mice were grouped randomly. The serum of mice was taken from tail vain, which were detected for HBsAg, HBeAg and HBV DNA levels regularly after injection. Serum HBsAg was measured by Time resolved fluoroimmunoassay (TRFIA) according to the manufacturer's instructions (Guangzhou DARUI Biotechnology Co.,Ltd). In order to adjust the values within the linear range of the standard curve, serum was 1:100 diluted by PBS. Serum HBeAg was measured by TRFIA with 1:20 dilution. And Real‐time qPCR was used to detect HBV DNA levels of model mice (Hunan Sansure Biotech Inc.). To collect liver tissues, mice were anesthetized using 1% Pentobarbital sodium. Liver tissues were preserved by 4% paraformaldehyde in PBS for ≈24 h, and then embedded in paraffin and sectioned (5 µm). Standard hematoxylin and eosin (H&E) and Masson's trichrome staining were used for pathological and immunohistochemical analyses by light microscopy. Besides, the mRNA levels of *Col1a1* and *Mmp2* were examined by real‐time PCR.

### Hepatocytes and Liver Nonparenchymal Cells Isolation

A two‐step collagenase perfusion procedure was applied to isolate hepatocytes from liver tissues. Briefly, liver was perfused to flush out blood and chelate calcium by cannulating the hepatic portal vein with a low flow rate gradually increasing to 7–9 ml min^−1^. The tissue became lighter with perfect perfusion for 10 min. Then pre‐warmed perfusion fluid containing 0.1%–0.15% of collagenase was injected to the liver in order to digest extracellular matrix for ≈10 min. Then, we turned off the pump and transferred the liver piece in a dish containing 5 ml pre‐warmed perfusion fluid with collagenase. The tissues were dissected out gently with removal of the gall bladder. Hepatocytes were released into suspension and subsequently isolated by centrifuging at 50 g for 5 min at 4 °C to separate from non‐hepatocyte cells.

To collect liver nonparenchymal cells, liver tissue samples were cut into pieces in pre‐cold DMEM, transferred into C tube and digested with mouse Liver Dissociation Kit (Miltenyi Biotec) for ≈40 min using a gentleMACS octo Dissociator with heaters according to the manufacturer's protocol. After dissociation, samples were resuspended and filtered by applying MACS smartStrainer (70 µm) with two times washing using DMEM. Then we used Debris Removal Solution (Miltenyi Biotec) to effectively remove cell debris from viable cells after dissociation of liver tissues during centrifugation, subsequently lysing of erythrocytes (RBC lysis buffer; Solarbio).

### Single‐Cell RNA Sequencing and Data Processing

Cells were isolated from liver tissues and loaded into the chromium controller (10X Genomics, Pleasanton, CA, USA) on single cell chip K or chip G. Single‐cell gel beads were generated following the manufacturer's protocol. The sequencing library construction was prepared using Single Cell 5′ or 3′ Library Kits, according to the 10X Chromium manufacturer's instruction. Libraries were sequenced by MGI‐2000H sequencer with 2 × 100 paired‐end kits using the following read length: 28 bp Read1 and 91 bp Read2 for 3′ Library kit and 26 bp Read1 and 90 bp Read2 for 5′ Library kit (Read1 for cell barcode and UMI and Read2 for transcript).

The Cell Ranger software pipeline (version 6.1.2) provided by 10X Genomics was used to demultiplex cell barcodes and map the reads against the mouse genome build (GRCm38). The unique molecular identifier (UMI) count matrix was integrated and processed using the R package Seurat (4.3.0). A stricter quality control was conducted, and the cells were filtered according to the following criteria: 1) the number of UMIs > 800. 2) number of genes > 500. 3) The percentage of mitochondria‐expressed genes < 10%, and for tissue Hepatocyte, the threshold is 40%. It was used DoubletFinder to estimate the doublet score with a 7.5% doublet formation rate. Cells with high doublet scores (0.2 as the cutoff) were discarded. After applying these quality control criteria, 115181 single cells in total were included in the downstream analyses.

### t‐SNE Analysis and Identification of Cell Types

The top 3000 highly variable genes for dimension reduction using the Seurat default parameters was identified. Principal component analysis was performed to reduce the dimensionality, and the top 30 principal components (PCs) were utilized to calculate the first two components of TSNE. A 1.0 resolution to identify clusters was used. Each cluster was annotated by their marker genes, and the clusters showing high expression of markers of two or more cell types were treated as doublets and removed. Finally, 10 cell types were identified for further analysis.

### Identification of DEGs and Enrichment Analysis

The “FindMarker” function in the Seurat package was employed to identify the DEGs between the HBV and control group with multiple threshold parameters: 1) average log2(fold change) ≥ 0.5, 2) *p* value < 0.05 and 3) detection in ≥ 10% of cells in at least one condition. GSEA enrichment were conducted using clusterProfiler R package (version 4.6.2) (Yu et al., 2012). Hallmark gene sets and ontology gene sets were obtained from MSigDB (https://www.gsea‐msgdb.org/gsea/msigdb/collection.jsp). Terms with an *p*‐adjust<0.05 were deemed statistically significant. The results were visualized using R software ggplot2 (version 3.4.2).

### Spatial Transcriptome Sequencing and Data Processing

Frozen liver tissues were embedded in pre‐chilled OCT (SAKURA Tissue‐Tek). The frozen samples were sectioned (10 µm) for downstream procedures (Cryostar NX70, ThermoFisher). The spacexr (version 0.1) package was utilized to map major cell types identified by scRNA‐seq in the profiled spatial transcriptomics sections. The Robust Cell Type Decomposition (RCTD) was invoked to estimate the abundance of each cell population in each location by decomposing mRNA counts in 10X Genomics Visium data using the transcriptional signatures of reference cell types. The standard RCTD analysis method was utilized in a doublet mode set to full.

### Digital Spatial Profiling Experiments and Data Processing

To explore the functional alteration of Kupffer cells in HBV transgenic mice, Nanostring GeoMx Digital Spatial Profiling (DSP) on autopsy FFPE slides (5 µm) was used. Slides were stained with Glutamine synthetase‐AF532 (Novus Bio, #NBP2‐70834AF532), F4/80‐AF647 (Abcam, #ab204467), and SYTO 13 (Thermo Fisher Scientific, #57 575) followed by fluorescent images scanning on the NanoString GeoMx instrument in the Alexa Fluor 532, Alexa Fluor 647, and SYTO 13 channels, respectively. Then transcriptomic profiling of selected regions of interest (ROIs) was obtained. Library preparation of each ROI was performed according to the manufacturer's instructions, followed by sequencing on a NovaSeq S2. DSP sequencing data was aggregated as previously described.^[^ ^77^ ^]^ Expression matrix was integrated, filtered, and normalized with upper quartile (Q3) normalization.

### Calculation of the Relative Colocalization Index

In order to validate whether the immune cells or chemokines were leaning toward CV rather than PV in spatial position after HBV infection, we defined the Relative Colocalization Index (RCI) to quantify the variation of spatial distribution. Taking Kupffer cells for example, the RCI of given spot *s* was defined as:

(1) $$\begin{array}{ccc} RCI\;\left(s\right) & = & \sum_{n\in nei\left(s\right)}ratio\;\left(KC,n\right)\;\;/\sum_{n\in nei\left(s\right)}ratio\;\left(CV,n\right)\\ & & -\sum_{n\in nei\left(s\right)}ratio\;\left(KC,n\right)/\sum_{n\in nei\left(s\right)}ratio\;\left(PV,n\right) \end{array}$$

ratio (cell type, *n*) was defined as the predicted proportion of given *cell* *type* in corresponding spot *n*. *nei*(*s*) was defined as the seven nearest spots of to spot *s* (including *s*). Only those spots with predicted proportion of both CV hep and PV hep > 0.1 were considered for the calculation. When RCI value in HBV model was higher than that in the control group, it indicated that Kupffer cells were localized more closely with CV under HBV infection. Wilcoxon‐test was performed for comparing, and the p‐value threshold was set to 0.05. RCI of other immune cells and chemokines were calculated in the same vein.

### Cellular Communication Networks Analysis

CellChat^[^ ^78^ ^]^ R package (https://github.com/sqjin/CellChat, accessed on 1 July 2022) was used to calculate, analyze, and infer intercellular communication from single‐cell transcriptional data. In the study, all of the cell types were extracted for intercellular communication analysis. The expression matrix of each cell types as input for CellChat to reveal significantly overexpressed ligand‐receptor pairs was set. Statistical tests were used to find significant interactions with *p* adjust value < 0.05. Moreover, CellChat to visualize the ligand‐receptor pair between Kupffer cells and PV/CV hepatocytes was used. The heatmap plot was performed to compare the communication strength by calculating the difference between HBV and Control groups at four time points.

### Multiplexed Immunofluorescence for Liver Sections

Multiplexed immunofluorescence labeling was performed on FFPE sections (5 µm). After de‐paraffinization and rehydration, Sodium citrate (pH6.0; Zsbio, ZLI‐9065) was used to perform antigen retrieval at 98 °C for 15 min. After blocking in 3% H_2_O_2_ (Zsbio, ZLI‐9311D) and normal Goat Serum (Zsbio, ZLI‐9056), liver sections were stained in primary antibodies overnight at 4 °C or 2 h at 37 °C. Sections were then incubated with secondary antibody (corresponding species, Zsbio) for 30 min at room temperature. Tyramide signal amplification (TSA) method was used for multiplex Immunohistochemistry (IHC). Cell nuclei were mounted with DAPI solution (Solarbio). The stained sections were imaged with LSM 880 fluorescence microscope (Carl Zeiss Meditec, Germany) or Nikon A1 confocal laser microscope (Nikon). If needed, images were analyzed subsequently using NIH ImageJ software v1.51.

When measuring CV‐dis and PV‐dis, images containing scale bars into ImageJ software was imported. The known length and unit of the scale bar were then entered and set within the software. Using the straight‐line tool, a line was drawn between the two points requiring measurement to obtain the distance. Specifically for this study, the distance from KCs (Kupffer cells, red regions) to the nearest CV (central vein, green region) or PV (portal vein, purple region) was measured. This represents the minimal distance between red and green regions as well as the minimal distance between red and purple regions.

Primary antibodies against Glutamine synthetase (1:1000, #ab73593), HBcAg (1:100, #ab8639) and HBsAg (1:400, #ab68520) were purchased from Abcam. CLEC4F (1:100, 370 901) was purchased from R&D systems (Minnesota, USA). F4/80 (1:300, 70 076) and CD31 (1:100, 77 699) antibody were purchased from Cell Signaling Technology (Beverly, MA, USA). E‐cadherin (1:1000, #50671‐RP02) was purchased from Sino Biological (Beijing, China). LXRα (1:200, #14352‐1‐AP), CXCL9 (1:200, #22355‐1‐AP), CXCR3 (1:200, #26756‐1‐AP), and NANOG (1:200, #14295‐1‐AP) antibodies were purchased from Proteintech (Wuhan, China)

### Spheroid Assay

HepAD38 cells were collected after co‐culturing with THP‐1 macrophages with or without LMD. Single cell suspensions were seeded into the 6‐well Ultra‐Low Attachment Microplates (Corning) at a density of 2 × 10^3^ cells well^−1^ to form spheroids. Then, the spheroids were cultured for up to 4 days. Moreover, the number and size of spheroids derived from HepAD38 cells were observed by EVOS M7000 imaging system (Invitrogen).

### Oil Red O Staining

After THP‐1 macrophages co‐cultured with HepAD38 in 6‐well plates had been treated, the cells were washed twice with PBS and fixed with 4% paraformaldehyde for 20 min. An oil red O dye solution (Beyotime) was used to perform the staining procedure. 60% isopropanol was used to rinse cells for 20 s. And then the cells were covered by oil red O dye for 15 min to be stained. The dye solution was removed followed by 60% isopropanol differentiation. Finally, the cells were washed with PBS and observed by EVOS M7000 imaging system (Invitrogen).

### siRNA Knockdown

Gene (including *CXCL9*, *ASGR1*, and *STAT3*)‐specific siRNA and nontargeting control siRNA were designed and synthesized by RiboBio (Guangzhou, China). The siRNA target sequences were as follows:

si*CXCL9*‐1: 5′‐ CCA AGG GAC TAT CCA CCT A ‐3′;
si*CXCL9*‐2: 5′‐ GTT CGA AAA TCT CAA CGT T ‐3′;
si*CXCL9*‐3: 5′‐ GGA GTT CAA ACA TGT CTA A ‐3′;
si*ASGR1*‐1: 5′‐ TGC TCC ACG TGA AGC AGT T ‐3′;
si*ASGR1*‐2: 5′‐ TGA CCA CCA TCA GCT CAG A ‐3′;
si*ASGR1*‐3: 5′‐ GAG GCA ATG TGG GAA GAA A ‐3′;
si*STAT3*‐1: 5′‐ GGC GTC CAG TTC ACT ACT A ‐3′;
si*STAT3*‐2: 5′‐ AGA CCC GTC AAC AAA TTA A ‐3′;
si*STAT3*‐3: 5′‐ CAT CGA GCA GCT GAC TAC A ‐3′;

*AKT*‐specific siRNA and miRNA‐specific inhibitor with negative control were designed and synthesized by Genepharma (Suzhou, China). The sequences of siRNAs were as follows:

si*AKT*‐1 sense: 5′‐ GCU ACU UCC UCC UCA AGA ATT ‐3′;
si*AKT*‐1 antisense: 5′‐ UUC UUG AGG AGG AAG UAG CTT ‐3′;
si*AKT*‐2 sense: 5′‐ CCA UGA AGA UCC UCA AGA ATT ‐3′;
si*AKT*‐2 antisense: 5′‐ UUC UUG AGG AUC UUC AUG GTT ‐3′;
si*AKT*‐3 sense: 5′‐ CGG AGA AGA ACG UGG UGU ATT ‐3′;
si*AKT*‐3 antisense: 5′‐ UAC ACC ACG UUC UUC UCC GTT ‐3′;
has‐miR‐155‐5p inhibitor: 5′‐ AAC CCC UAU CAC GAU UAG CAU UAA ‐3′;
has‐miR‐206 inhibitor: 5′‐ CCA CAC ACU UCC UUA CAU UCC A ‐3′;
miR‐inhibitor negative control: 5′‐ CAG UAC UUU UGU GUA GUA CAA ‐3′.

Those siRNA and miRNA‐specific inhibitor were transfected into cells by using CALNP RNAi in vitro (D‐Nano, #DN001‐05) following manufacturer's instructions.

### RNA Extraction and Quantitative PCR

Total RNA was extracted from cells or liver tissues using lysis buffer from RNApure Tissue & Cell Kit (DNase I) (#CW0560, Cwbio). Reverse transcription was performed with Fasking gDNA Dispelling RT SuperMix (#KR118, Tiangen). Real‐time PCR was performed using QuantiNova SYBR Green PCR kit (Qiagen) and detected on 7500 Real‐Time PCR System (Applied Biosystems). RNA expression levels were calculated based on the ΔΔCt method.

### Western Blotting

Cell lysates were prepared in RIPA lysis buffer (P0013B, Beyotime). Sodium dodecyl sulfate–polyacrylamide gel electrophoresis (SDS‐PAGE) was used to separate proteins of different molecular weights. Then proteins were transferred to polyvinylidene difluoride (PVDF) membranes through a trans‐blot cell system (Bio‐Rad). The PVDF Membranes were blocked with 5% (w/v) skimmed milk powder in TBS‐T, followed by incubation in primary antibodies and corresponding secondary antibodies. Blots were presented using enhanced chemiluminescent (ECL) detection system.

Primary antibodies against LXRα (#14352‐1‐AP), ASGR1 (#11739‐1‐AP), BRCA1 (#22362‐1‐AP), BARD1 (#22964‐1‐AP), NANOG (#14295‐1‐AP), LIN28 (#11724‐1‐AP), OCT4 (#11263‐1‐AP), STAT3 (#10253‐1‐AP), WNT3A (#26744‐1‐AP), and CXCL9 (#22355‐1‐AP), were purchased from Proteintech (Wuhan, China). ABCA1 (#96 292), *p*‐STAT3 (#9145), *p*‐AKT (#4060) and AKT (#4691) antibodies were purchased from Cell Signaling Technology (Beverly, MA, USA). The antibody clone T2221 (#2AHC24) specific for HBc N‐terminal domain was purchased from Tokyo Future Style.

### Immunoprecipitation

To investigate the impact of ASGR1 on LXRα ubiquitination, we performed co‐immunoprecipitation (co‐IP) assays as follows: THP‐1‐derived macrophages were first transfected with siASGR1 (or negative control) and incubated at 37 °C for 24 h to suppress ASGR1 expression. Subsequently, these cells were co‐cultured with HepAD38 cells (tet‐off or tet‐on) for an additional 48 h. To preserve ubiquitinated LXRα species, cells were treated with 10 µM MG132 for 6 h prior to lysis. Cell lysates were prepared using RIPA buffer supplemented with protease and phosphatase inhibitors, then incubated overnight at 4 °C with anti‐LXRα antibody‐conjugated agarose beads (Pierce Magnetic IP/Co‐IP Kit, Thermo Fisher Scientific, #88 804) to immunoprecipitate LXRα and its associated interactors. After thorough washing, both the input (10% of total lysate) and bead‐bound (IP) fractions were subjected to SDS‐PAGE, transferred to PVDF membranes, and probed with anti‐LXRα, anti‐ubiquitin (Proteintech, #1020‐1‐2‐AP), and anti‐*β*‐actin antibodies.

### Flow Cytometry and Intracellular Cytokine Staining

NPCs from liver were isolated with the two‐step collagenase perfusion procedure mentioned above. To examine the expression levels of Cxcl9 in various endothelial cell types, cell suspensions were incubated for 4–6 h in the presence of brefeldin A solution (#420 601, Biolegend) and monensin solution (#420 701, Biolegend). Then, cell suspensions were stained with fluorophore‐conjugated antibodies purchased from Biolegend (San Diego, USA) to the following mouse cell‐surface markers for 30 min: Cd45‐Alexa Fluor 700 (#109 822), Cd31‐BV421 (#102 423), Cd204‐APC (#145 711), and Cd117‐PE‐Cy7 (#105 813), followed by incubation in Fixation/Permeabilization solution (BD biosciences) for 20 min at 4 °C. Finally, cells were stained with Cxcl9‐PE antibody (#515 603). Besides, CD133‐PE antibody (#394 003), CD326‐Alexa Fluor 488 antibody (#324 209), CD90 (Thy1)‐APC‐Cy7 antibody (#328 131) from Biolegend were used to detect cell stemness of HepAD38 cell line. Flow cytometry was performed using Sony ID7000 Spectral Cell Analyzer (Sony Biotechnology Inc., USA) and analyzed using FlowJo 10.5.0 software.

### Southern Blotting of viral DNA

HepAD38 cells were cultured without tetracycline for 1 week to accumulate viral DNA and acted as positive control. Replicative viral DNA intermediates were purified from cytoplasmic core particles of HepAD38 lysates. Cells were lysed in NP‐40 buffer and the cytoplasmic lysate (supernatant) was obtained after removal of the nuclear pellet by centrifugation. Micrococcal nuclease (MNase, Roche) and CaCl_2_ were used to degrade the nucleic acids outside nucleocapsids, which was terminated by addition of EDTA. SDS (0.6%)‐proteinase K (0.5 mg mL^−1^) digestion was performed to remove all proteins and DNA was purified by phenol‐chloroform ethanol precipitation. Purified HBV DNA was resolved on a 1.2% agarose gel, transferred onto a nitrocellulose membrane, and hybridized with a ^32^P‐labled HBV DNA probe as previously described.^[^ ^79^, ^80^ ^]^

### HBV Capsid Assembly Analysis

To determine the levels of assembled capsids, cytoplasmic lysates were resolved on 1% native agarose gels (native agarose gel electrophoresis or NAGE). Upon transferring the resolved capsids onto a nitrocellulose membrane, capsids were detected by using the indicated HBc antibody.

### Inflammation‐Related Biomarkers Measurement

Supernatant samples from co‐cultured systems (Normal: *n* = 3; Normal_GW: *n* = 3; LMD: *n* = 3; LMD_GW: *n* = 3) were analyzed using Olink Inflammation multiplex platform (Uppsala, Sweden), which enables 92 soluble analytes to be analyzed simultaneously based on the Proximity Extension Assay (PEA) technology.^[^ ^31^ ^]^ Briefly, each soluble analyte was recognized by double antibodies coupled with oligonucleotides which were complementary. The levels of analyte‐specific oligonucleotide were quantified using a high‐throughput instrument (Fluidigm Biomark HD; South San Francisco, CA). The normalized protein level was calculated on the concentration of oligonucleotide.

### Statistics Analysis

Multiple statistical methods were described above. Potentially variable results were repeated to ensure reproducibility. The statistical analysis between two groups was performed and analyzed by two‐tailed student's *t*‐test in GraphPad Prism 9.0 software. One‐way ANOVA or Two‐way ANOVA followed by Tukey's post hoc test was used to compare multiple groups. Data were shown as mean ± Standard Error of the Mean (SEM). An adjusted *p*‐value < 0.05 was generally used as a threshold for statistical significance. *p<0.05, **p<0.01, ***p<0.001, ****p<0.0001

## Conflict of Interest

The authors declare no conflict of interest.

## Author Contributions

J.S., Q.L., and J.L. contributed equally to this work. S.W., L.G., W. S., and J.S. conceived, designed, and interpreted the experiments. L.G., J.S., Q.L., and J.L. performed the experiments and wrote the manuscript with input from other authors. J.B., J.X., X.L., and X.W. contributed to the experiments associated with HBV infection and detection. Q.L., J.Z., and X.Z. developed the pipeline for scRNA‐seq and ST data analysis. Q.H., X.S, and Y.C. performed the immunohistochemical staining and image analysis. Z.S., X.Y., J.L. and W.Y. performed the library construction and sequencing.

## Supporting information



Supporting Information

Supporting Tables

## Data Availability

The data that support the findings of this study were uploaded on the Genome Sequence Archive in China National GeneBank DataBase (CNGBdb) accessible at https://db.cngb.org/ (CNP0006059). Original code has been uploaded at github (https://github.com/QingyuLiaib/HBV_scRNA).

## References

[1] W.‐K. Seto , Y.‐R. Lo , J.‐M. Pawlotsky , M.‐F. Yuen , Lancet 2018, 392, 2313. 30496122 10.1016/S0140-6736(18)31865-8

[2] Y.‐C. Hsu , D. Q. Huang , M. H. Nguyen , Nat. Rev. Gastroenterol. Hepatol. 2023, 20, 524. 37024566 10.1038/s41575-023-00760-9

[3] E. P. Tsounis , E. Tourkochristou , A. Mouzaki , C. Triantos , World J. Gastroenterol. 2021, 27, 2727. 34135551 10.3748/wjg.v27.i21.2727 PMC8173382

[4] W. Leowattana , T. Leowattana , World J. Virol. 2022, 11, 57. 35117971 10.5501/wjv.v11.i1.57 PMC8788212

[5] C. Le , Y. Liu , J. López‐Orozco , M. A. Joyce , X. C. Le , D. L. Tyrrell , Anal. Chem. 2021, 93, 10756. 34328316 10.1021/acs.analchem.1c02227

[6] A. Khanam , J. V. Chua , S. Kottilil , Int. J. Mol. Sci. 2021, 22, 5497. 34071064 10.3390/ijms22115497 PMC8197097

[7] K. B. Halpern , R. Shenhav , O. Matcovitch‐Natan , B. Tóth , D. Lemze , M. Golan , E. E. Massasa , S. Baydatch , S. Landen , A. E. Moor , A. Brandis , A. Giladi , A. Stokar‐Avihail , E. David , I. Amit , S. Itzkovitz , Nature 2017, 542, 352. 28166538 10.1038/nature21065 PMC5321580

[8] S. A. MacParland , J. C. Liu , X.‐Z. Ma , B. T. Innes , A. M. Bartczak , B. K. Gage , J. Manuel , N. Khuu , J. Echeverri , I. Linares , R. Gupta , M. L. Cheng , L. Y. Liu , D. Camat , S. W. Chung , R. K. Seliga , Z. Shao , E. Lee , S. Ogawa , M. Ogawa , M. D. Wilson , J. E. Fish , M. Selzner , A. Ghanekar , D. Grant , P. Greig , G. Sapisochin , N. Selzner , N. Winegarden , O. Adeyi , et al., Nat. Commun. 2018, 9, 4383. 30348985 10.1038/s41467-018-06318-7 PMC6197289

[9] V. L. Payen , A. Lavergne , N. Alevra Sarika , M. Colonval , L. Karim , M. Deckers , M. Najimi , W. Coppieters , B. Charloteaux , E. M. Sokal , A. El Taghdouini , JHEP Rep. Innov. Hepatol. 2021, 3, 100278. 10.1016/j.jhepr.2021.100278 PMC8121977 34027339

[10] J. Zhao , S. Zhang , Y. Liu , X. He , M. Qu , G. Xu , H. Wang , M. Huang , J. Pan , Z. Liu , Z. Li , L. Liu , Z. Zhang , Cell Discov. 2020, 6, 22. 32351704 10.1038/s41421-020-0157-z PMC7186229

[11] P. Ramachandran , R. Dobie , J. R. Wilson‐Kanamori , E. F. Dora , B. E. P. Henderson , N. T. Luu , J. R. Portman , K. P. Matchett , M. Brice , J. A. Marwick , R. S. Taylor , M. Efremova , R. Vento‐Tormo , N. O. Carragher , T. J. Kendall , J. A. Fallowfield , E. M. Harrison , D. J. Mole , S. J. Wigmore , P. N. Newsome , C. J. Weston , J. P. Iredale , F. Tacke , J. W. Pollard , C. P. Ponting , J. C. Marioni , S. A. Teichmann , N. C. Henderson , Nature 2019, 575, 512. 31597160 10.1038/s41586-019-1631-3 PMC6876711

[12] G. Song , Y. Shi , M. Zhang , S. Goswami , S. Afridi , L. Meng , J. Ma , Y. Chen , Y. Lin , J. Zhang , Y. Liu , Z. Jin , S. Yang , D. Rao , S. Zhang , A. Ke , X. Wang , Y. Cao , J. Zhou , J. Fan , X. Zhang , R. Xi , Q. Gao , Cell Discov. 2020, 6, 90. 33298893 10.1038/s41421-020-00214-5 PMC7721904

[13] O. Krenkel , F. Tacke , Nat. Rev. Immunol. 2017, 17, 306. 28317925 10.1038/nri.2017.11

[14] F. Yuan , W. Zhang , D. Mu , J. Gong , Adv. Clin. Exp. Med. Off. Organ Wroclaw Med. Univ. 2017, 26, 739. 10.17219/acem/62759 28691411

[15] M. Peiseler , B. Araujo David , J. Zindel , B. G. J. Surewaard , W.‐Y. Lee , F. Heymann , Y. Nusse , F. V. S. Castanheira , R. Shim , A. Guillot , A. Bruneau , J. Atif , C. Perciani , C. Ohland , P. Ganguli Mukherjee , A. Niehrs , R. Thuenauer , M. Altfeld , M. Amrein , Z. Liu , P. M. K. Gordon , K. McCoy , J. Deniset , S. MacParland , F. Ginhoux , F. Tacke , P. Kubes , Science 2023, 381, abq5202. 10.1126/science.abq5202 37676943

[16] L. He , W. Pu , X. Liu , Z. Zhang , M. Han , Y. Li , X. Huang , X. Han , Y. Li , K. Liu , M. Shi , L. Lai , R. Sun , Q.‐D. Wang , Y. Ji , J. S. Tchorz , B. Zhou , Science 2021, 371, abc4346. 10.1126/science.abc4346 33632818

[17] J. Paris , N. C. Henderson , Hepatology 2022, 76, 1219. 35175659 10.1002/hep.32408 PMC9790419

[18] S. Ben‐Moshe , S. Itzkovitz , Nat. Rev. Gastroenterol. Hepatol. 2019, 16, 395. 30936469 10.1038/s41575-019-0134-x

[19] A. Saviano , N. C. Henderson , T. F. Baumert , J. Hepatol. 2020, 73, 1219. 32534107 10.1016/j.jhep.2020.06.004 PMC7116221

[20] A. Gola , M. G. Dorrington , E. Speranza , C. Sala , R. M. Shih , A. J. Radtke , H. S. Wong , A. P. Baptista , J. M. Hernandez , G. Castellani , I. D. C. Fraser , R. N. Germain , Nature 2021, 589, 131. 33239787 10.1038/s41586-020-2977-2 PMC8691525

[21] M. Guilliams , J. Bonnardel , B. Haest , B. Vanderborght , C. Wagner , A. Remmerie , A. Bujko , L. Martens , T. Thoné , R. Browaeys , F. F. De Ponti , B. Vanneste , C. Zwicker , F. R. Svedberg , T. Vanhalewyn , A. Gonçalves , S. Lippens , B. Devriendt , E. Cox , G. Ferrero , V. Wittamer , A. Willaert , S. J. F. Kaptein , J. Neyts , K. Dallmeier , P. Geldhof , S. Casaert , B. Deplancke , P. ten Dijke , A. Hoorens , et al., Cell 2022, 185, 379. 35021063 10.1016/j.cell.2021.12.018 PMC8809252

[22] A. Guillot , M. Winkler , M. Silva Afonso , A. Aggarwal , D. Lopez , H. Berger , M. S. Kohlhepp , H. Liu , B. Özdirik , J. Eschrich , J. Ma , M. Peiseler , F. Heymann , S. Pendem , S. Mahadevan , B. Gao , L. Diehl , R. Gupta , F. Tacke , Hepatol. Baltim. Md 2023, 78, 150. 10.1097/HEP.0000000000000270 36630995

[23] D. Zhao , F. Yang , Y. Wang , S. Li , Y. Li , F. Hou , W. Yang , D. Liu , Y. Tao , Q. Li , J. Wang , F. He , L. Tang , J. Clin. Invest. 2022, 132, 150489. 10.1172/JCI150489 PMC8803331 34874921

[24] J. Lucifora , A. Salvetti , X. Marniquet , L. Mailly , B. Testoni , F. Fusil , A. Inchauspé , M. Michelet , M.‐L. Michel , M. Levrero , P. Cortez , T. F. Baumert , F.‐L. Cosset , C. Challier , F. Zoulim , D. Durantel , Antiviral Res. 2017, 145, 14. 28709657 10.1016/j.antiviral.2017.07.006

[25] C. Xu , J. Fan , D. Liu , A. Tuerdi , J. Chen , Y. Wei , Y. Pan , H. Dang , X. Wei , A. S. Yousif , J. Yogaratnam , Q. Zhou , H. Lichenstein , T. Xu , Hepatol. Baltim. Md 2023, 77, 275. 10.1002/hep.32614 35699669

[26] F. Kan , L. Ye , T. Yan , J. Cao , J. Zheng , W. Li , BMC Genomics 2017, 18, 641. 28830339 10.1186/s12864-017-3984-z PMC5568174

[27] Y.‐H. Huang , C.‐C. Fang , K. Tsuneyama , H.‐Y. Chou , W.‐Y. Pan , Y.‐M. Shih , P.‐Y. Wu , Y. Chin , P. S. C. Leung , M. Eric Gershwin , M.‐H. Tao , Int. J. Oncol. 2011, 39, 1511 . 21805030 10.3892/ijo.2011.1145

[28] Z. Liao , C. Tang , R. Luo , X. Gu , J. Zhou , J. Gao , Diagnostics 2023, 13, 1211. 37046429 10.3390/diagnostics13071211 PMC10093043

[29] P. Hytiroglou , Y. N. Park , G. Krinsky , N. Theise , Gastroenterol. Clin. North Am. 2008, 36, 867. 10.1016/j.gtc.2007.08.010 17996795

[30] D. M. Cable , E. Murray , L. S. Zou , A. Goeva , E. Z. Macosko , F. Chen , R. A. Irizarry , Nat. Biotechnol. 2022, 40, 517. 33603203 10.1038/s41587-021-00830-w PMC8606190

[31] M. Ringelhan , U. Protzer , Curr. Opin. Virol. 2015, 14, 109. 26426688 10.1016/j.coviro.2015.08.015

[32] N. Zelcer , P. Tontonoz , J. Clin. Invest. 2006, 116, 607. 16511593 10.1172/JCI27883 PMC1386115

[33] N. Wang , S. Wang , M.‐Y. Li , B.‐G. Hu , L.‐P. Liu , S.‐L. Yang , S. Yang , Z. Gong , P. B. S. Lai , G. G. Chen , Ther. Adv. Med. Oncol. 2018, 10, 1758835918816287. 30622654 10.1177/1758835918816287 PMC6304707

[34] T. Yamashita , X. W. Wang , J. Clin. Invest. 2013, 123, 1911. 23635789 10.1172/JCI66024 PMC3635728

[35] P. Vasefifar , R. Motafakkerazad , L. A. Maleki , S. Najafi , F. Ghrobaninezhad , B. Najafzadeh , H. Alemohammad , M. Amini , A. Baghbanzadeh , B. Baradaran , Gene 2022, 827, 146448. 35337852 10.1016/j.gene.2022.146448

[36] Y.‐C. Liu , C.‐T. Yeh , K.‐H. Lin , Cells 2020, 9, 1331.32466488

[37] L. S. Ali , Y. A. M. Attia , S. Mourad , E. M. Halawa , N. H. Abd Elghaffar , S. Shokry , O. M. Attia , M. Makram , A.‐H. S. Wadan , W. A. Negm , E. Elekhnawy , Curr. Med. Res. Opin. 2024, 40, 1963. 39316769 10.1080/03007995.2024.2407963

[38] L. Guo , C. Chen , M. Shi , F. Wang , X. Chen , D. Diao , M. Hu , M. Yu , L. Qian , N. Guo , Oncogene 2013, 32, 5272. 23318420 10.1038/onc.2012.573

[39] P. Xia , X.‐Y. Xu , Am. J. Cancer Res. 2015, 5, 1602. 26175931 PMC4497429

[40] T. Reya , H. Clevers , Nature 2005, 434, 843. 15829953 10.1038/nature03319

[41] C. Buccitelli , M. Selbach , Nat. Rev. Genet. 2020, 21, 630. 32709985 10.1038/s41576-020-0258-4

[42] J.‐Q. Wang , L.‐L. Li , A. Hu , G. Deng , J. Wei , Y.‐F. Li , Y.‐B. Liu , X.‐Y. Lu , Z.‐P. Qiu , X.‐J. Shi , X. Zhao , J. Luo , B.‐L. Song , Nature 2022, 608, 413. 35922515 10.1038/s41586-022-05006-3

[43] L. Ye , H. Yu , C. Li , M. L. Hirsch , L. Zhang , R. J. Samulski , W. Li , Z. Liu , PLoS One 2015, 10, 0130052. 10.1371/journal.pone.0130052 PMC4468063 26075890

[44] D. Yang , L. Liu , D. Zhu , H. Peng , L. Su , Y.‐X. Fu , L. Zhang , Cell. Mol. Immunol. 2014, 11, 71. 24076617 10.1038/cmi.2013.43 PMC4002146

[45] J. L. Baratta , A. Ngo , B. Lopez , N. Kasabwalla , K. J. Longmuir , R. T. Robertson , Histochem. Cell Biol. 2009, 131, 713. 19255771 10.1007/s00418-009-0577-1 PMC2761764

[46] K. Ray , Nat. Rev. Gastroenterol. Hepatol. 2021, 18, 81. 33335281 10.1038/s41575-020-00403-3

[47] S. A. Means , M. A. Ali , H. Ho , Front. Syst. Biol. 2023, 3, 1045754. 40809484 10.3389/fsysb.2023.1045754 PMC12341981

[48] P. A. C. Wing , P. J. Liu , J. M. Harris , A. Magri , T. Michler , X. Zhuang , H. Borrmann , R. Minisini , N. R. Frampton , J. M. Wettengel , L. Mailly , V. D'Arienzo , T. Riedl , L. Nobre , M. P. Weekes , M. Pirisi , M. Heikenwalder , T. F. Baumert , E. M. Hammond , D. R. Mole , U. Protzer , P. Balfe , J. A. McKeating , J. Hepatol. 2021, 75, 64. 33516779 10.1016/j.jhep.2020.12.034 PMC8214165

[49] K. B. Halpern , R. Shenhav , H. Massalha , B. Toth , A. Egozi , E. E. Massasa , C. Medgalia , E. David , A. Giladi , A. E. Moor , Z. Porat , I. Amit , S. Itzkovitz , Nat. Biotechnol. 2018, 36, 962. 30222169 10.1038/nbt.4231 PMC6546596

[50] T. S. Andrews , J. Atif , J. C. Liu , C. T. Perciani , X.‐Z. Ma , C. Thoeni , M. Slyper , G. Eraslan , A. Segerstolpe , J. Manuel , S. Chung , E. Winter , I. Cirlan , N. Khuu , S. Fischer , O. Rozenblatt‐Rosen , A. Regev , I. D. McGilvray , G. D. Bader , S. A. MacParland , Hepatol. Commun. 2022, 6, 821. 34792289 10.1002/hep4.1854 PMC8948611

[51] M. Kurowska‐Stolarska , M. K. Hasoo , D. J. Welsh , L. Stewart , D. McIntyre , B. E. Morton , S. Johnstone , A. M. Miller , D. L. Asquith , N. L. Millar , A. B. Millar , C. A. Feghali‐Bostwick , N. Hirani , P. J. Crick , Y. Wang , W. J. Griffiths , I. B. McInnes , C. McSharry , J. Allergy Clin. Immunol. 2017, 139, 1946. 27746237 10.1016/j.jaci.2016.09.021 PMC5457127

[52] M. Vinod , I. Chennamsetty , S. Colin , L. Belloy , F. De Paoli , H. Schaider , W. F. Graier , S. Frank , D. Kratky , B. Staels , G. Chinetti‐Gbaguidi , G. M. Kostner , Biochim. Biophys. Acta 2014, 1841, 827. 24603323 10.1016/j.bbalip.2014.02.006 PMC3996726

[53] D. Zhong , G. Huang , Y. Zhang , Y. Zeng , Z. Xu , Y. Zhao , X. He , F. He , Cell. Signal. 2013, 25, 1429. 23499676 10.1016/j.cellsig.2013.03.003

[54] C. L. Scott , F. Zheng , P. De Baetselier , L. Martens , Y. Saeys , S. De Prijck , S. Lippens , C. Abels , S. Schoonooghe , G. Raes , N. Devoogdt , B. N. Lambrecht , A. Beschin , M. Guilliams , Nat. Commun. 2016, 7, 10321. 26813785 10.1038/ncomms10321 PMC4737801

[55] C. L. Scott , M. Guilliams , J. Hepatol. 2018, 69, 1197. 30001821 10.1016/j.jhep.2018.02.013 PMC7611037

[56] M.‐S. Lee , S. J. Bensinger , Cell. Mol. Immunol. 2022, 19, 327. 35017717 10.1038/s41423-021-00827-0 PMC8891295

[57] J. Yan , T. Horng , Trends Cell Biol. 2020, 30, 979. 33036870 10.1016/j.tcb.2020.09.006

[58] C. L. Scott , W. T'Jonck , L. Martens , H. Todorov , D. Sichien , B. Soen , J. Bonnardel , S. De Prijck , N. Vandamme , R. Cannoodt , W. Saelens , B. Vanneste , W. Toussaint , P. De Bleser , N. Takahashi , P. Vandenabeele , S. Henri , C. Pridans , D. A. Hume , B. N. Lambrecht , P. De Baetselier , S. W. F. Milling , J. A. Van Ginderachter , B. Malissen , G. Berx , A. Beschin , Y. Saeys , M. Guilliams , Immunity 2018, 49, 312. 30076102 10.1016/j.immuni.2018.07.004 PMC6104815

[59] M. Sakai , T. D. Troutman , J. S. Seidman , Z. Ouyang , N. J. Spann , Y. Abe , K. M. Ego , C. M. Bruni , Z. Deng , J. C. M. Schlachetzki , A. Nott , H. Bennett , J. Chang , B. T. Vu , M. P. Pasillas , V. M. Link , L. Texari , S. Heinz , B. M. Thompson , J. G. McDonald , F. Geissmann , C. K. Glass , Immunity 2019, 51, 655. 31587991 10.1016/j.immuni.2019.09.002 PMC6800814

[60] P. A. Louwe , M. Guilliams , Science 2023, 381, 1050. 37676940 10.1126/science.adj9725

[61] S. M. Afify , A. Sanchez Calle , G. Hassan , K. Kumon , H. M. Nawara , M. H. Zahra , H. M. Mansour , A. C. Khayrani , Md. Alam , J. Du , A. Seno , Y. Iwasaki , M. Seno , Br. J. Cancer 2020, 122, 1378. 32203212 10.1038/s41416-020-0792-z PMC7188674

[62] K. Cheng , N. Cai , J. Zhu , X. Yang , H. Liang , W. Zhang , Cancer Commun. Lond. Engl. 2022, 42, 1112. 10.1002/cac2.12345 PMC9648394 36069342

[63] D. Chen , X. Zhang , Z. Li , B. Zhu , Theranostics 2021, 11, 1016. 33391518 10.7150/thno.51777 PMC7738889

[64] M. Song , J. He , Q.‐Z. Pan , J. Yang , J. Zhao , Y.‐J. Zhang , Y. Huang , Y. Tang , Q. Wang , J. He , J. Gu , Y. Li , S. Chen , J. Zeng , Z.‐Q. Zhou , C. Yang , Y. Han , H. Chen , T. Xiang , D.‐S. Weng , J.‐C. Xia , Hepatology 2021, 73, 1717. 33682185 10.1002/hep.31792

[65] G. Cui , Z. Wang , H. Liu , Z. Pang , Front. Immunol. 2022, 13, 1057181. 36466926 10.3389/fimmu.2022.1057181 PMC9714270

[66] V. Salemme , G. Centonze , F. Cavallo , P. Defilippi , L. Conti , Front. Oncol. 2021, 11, 610303. 33777750 10.3389/fonc.2021.610303 PMC7991834

[67] J. Zhang , J. Guo , N. Yang , Y. Huang , T. Hu , C. Rao , Cell Death Dis. 2022, 13, 1051. 36535923 10.1038/s41419-022-05444-x PMC9763476

[68] J. Bonnardel , W. T'Jonck , D. Gaublomme , R. Browaeys , C. L. Scott , L. Martens , B. Vanneste , S. De Prijck , S. A. Nedospasov , A. Kremer , E. Van Hamme , P. Borghgraef , W. Toussaint , P. De Bleser , I. Mannaerts , A. Beschin , L. A. van Grunsven , B. N. Lambrecht , T. Taghon , S. Lippens , D. Elewaut , Y. Saeys , M. Guilliams , Immunity 2019, 51, 638. 31561945 10.1016/j.immuni.2019.08.017 PMC6876284

[69] E. Mass , I. Ballesteros , M. Farlik , F. Halbritter , P. Günther , L. Crozet , C. E. Jacome‐Galarza , K. Händler , J. Klughammer , Y. Kobayashi , E. Gomez‐Perdiguero , J. L. Schultze , M. Beyer , C. Bock , F. Geissmann , Science 2016, 353, aaf4238. 27492475 10.1126/science.aaf4238 PMC5066309

[70] H. Li , Y. Zhou , H. Wang , M. Zhang , P. Qiu , M. Zhang , R. Zhang , Q. Zhao , J. Liu , Front. Immunol. 2020, 11, 1169. 32670278 10.3389/fimmu.2020.01169 PMC7326822

[71] S.‐B. Su , S.‐Y. Qin , X.‐L. Xian , F.‐F. Huang , Q.‐L. Huang , H.‐J. ZhangDi , H.‐X. Jiang , Life Sci. 2021, 264, 118677. 33129875 10.1016/j.lfs.2020.118677

[72] Z. Yu , X. Xie , X. Su , H. Lv , S. Song , C. Liu , Y. You , M. Tian , L. Zhu , L. Wang , J. Qi , Q. Zhu , Cell. Signal. 2022, 93, 110304. 35278669 10.1016/j.cellsig.2022.110304

[73] D. J. Kloosterman , J. Erbani , M. Boon , M. Farber , S. M. Handgraaf , M. Ando‐Kuri , E. Sánchez‐López , B. Fontein , M. Mertz , M. Nieuwland , N. Q. Liu , G. Forn‐Cuni , N. N. van der Wel , A. E. Grootemaat , L. Reinalda , S. I. van Kasteren , E. de Wit , B. Ruffell , E. Snaar‐Jagalska , K. Petrecca , D. Brandsma , A. Kros , M. Giera , L. Akkari , Cell 2024, 187, 5336. 39137777 10.1016/j.cell.2024.07.030 PMC11429458

[74] M. B. Fessler , Pharmacol. Ther. 2018, 181, 1. 28720427 10.1016/j.pharmthera.2017.07.010 PMC5743771

[75] T. G. Kirchgessner , P. Sleph , J. Ostrowski , J. Lupisella , C. S. Ryan , X. Liu , G. Fernando , D. Grimm , P. Shipkova , R. Zhang , R. Garcia , J. Zhu , A. He , H. Malone , R. Martin , K. Behnia , Z. Wang , Y. Barrett , R. J. Garmise , L. Yuan , J. Zhang , M. D. Gandhi , P. Wastall , T. Li , S. Du , L. Salvador , R. Mohan , G. H. Cantor , E. Kick , J. Lee , Cell Metab. 2016, 24, 223. 27508871 10.1016/j.cmet.2016.07.016

[76] A. Katz , C. Udata , E. Ott , L. Hickey , M. E. Burczynski , P. Burghart , O. Vesterqvist , X. Meng , J. Clin. Pharmacol. 2009, 49, 643. 19398602 10.1177/0091270009335768

[77] C. R. Merritt , G. T. Ong , S. E. Church , K. Barker , P. Danaher , G. Geiss , M. Hoang , J. Jung , Y. Liang , J. McKay‐Fleisch , K. Nguyen , Z. Norgaard , K. Sorg , I. Sprague , C. Warren , S. Warren , P. J. Webster , Z. Zhou , D. R. Zollinger , D. L. Dunaway , G. B. Mills , J. M. Beechem , Nat. Biotechnol. 2020, 38, 586. 32393914 10.1038/s41587-020-0472-9

[78] S. Jin , C. F. Guerrero‐Juarez , L. Zhang , I. Chang , R. Ramos , C.‐H. Kuan , P. Myung , M. V. Plikus , Q. Nie , Nat. Commun. 2021, 12, 1088. 33597522 10.1038/s41467-021-21246-9 PMC7889871

[79] J. Xi , L. Luckenbaugh , J. Hu , PLoS Pathog. 2021, 17, 1009230. 10.1371/journal.ppat.1009230 PMC7861550 33493210

[80] J. Luo , X. Cui , L. Gao , J. Hu , J. Virol. 2017, 91, 00539. 10.1128/JVI.00539-17 PMC5553186 28637752

