# Supplementary material for: CHB‐Induced Immune Zonation Chaos Elicited LXRα‐mediated Lipid Metabolism Disorders in Kupffer Cells to Induce Cancer Stem Cell Formation
Source: Adv Sci (Weinh). 2025 Oct 30;13(10):e10275. doi: 10.1002/advs.202510275 (PMC12915127; doi:10.1002/advs.202510275)
Supplement: Supplementary file 1 — Supporting Information [file ADVS-13-e10275-s002.doc]

**Figure S1.** **AAV-HBV mouse models delineated the natural history of CHB progression.**

(a) Persistent infection of HBV demonstrated by a sustained high level of HBsAg, HBeAg, and HBV DNA in the serum of AAV-HBV mice at different time points. (n = 3-10). (b) The average body weight of at lease three mice from each control at indicated time points. (n = 3-9). (c) Masson’s Trichrome (M&T) staining of collagen fibers (left) and the mRNA level of fibrosis biomarkers (right) of liver tissue samples derived from the control and AAV-HBV mouse models at three, six and twelve months after intravenous injection (n = 3). (d) The serum concentration of AFP was significantly elevated at 12 months post infection by ELISA assay (n = 3). (e) The representative image of small cell change (SCC) in the H&E staining of liver FFPE sections from the 12-month CHB mice and control mice. (f) The representative images of the macroscopic livers and the M&T and H&E staining of the liver FFPE sections from the 27-month of CHB mice. (g-h) The violin plots of the distribution number of UMIs (left), detected genes (middle) and the percentage of mitochondria-expressed genes (right) per cell for hepatocytes (g) and non-parenchymal cells (h). (i) T-SNE plots of the parenchymal and non-parenchymal cell types. Each cell type cluster was color-coded via the canonical signature gene expression. (j) The violin plot of the distribution number of UMIs (upper) and the detected genes (lower) per plot for the Visium data. (k) Significantly increased level of *Afp* in the sample of the 27-month of CHB mice by the Visium transcriptomics data. Data are shown as mean ± SEM; **p*<0.05, ***p*<0.01, ****p*<0.001, *****p*<0.0001

**Figure S2. The relative colocalization index (RCI) of NPCs upon prolonged HBV infection.**

(a) The dynamic RCI variations of B cell, T cell, NK cell, dendritic cell, Macrophage/Monocytes, and neutrophils in the AAV-HBV and control mice at indicated time points by the Visium data. (b) The spatial distribution of Kupffer cells and peri-central hepatocytes (CV hep) within the Visium plots of the livers of the control and CHB mice models of different time points. (c) The dynamic cellular communication network analysis of the potential crosstalk between different cell types with the progression of the disease. A consistently higher score of the hepatocyte-KC interaction was revealed in CHB mice at all time points. (d) The variation pattern of the ratio of the distance between KC and PV to that of KC and CV (PV Distance/CV Distance). Three field-of-view of the mIF images from the control and CHB mice of indicated time points were selected for statistical analysis. (e) Visualization of the each measured distance between KCs and central vein (CV) area upon long-term of CHB infection. Each measured distance of individual KCs to the nearest central vein (CV Distance) was examined on three selected field-of-view of the mIF images from each group at indicated time points. (n = 3 biological independent samples). Data shown are mean ± SEM; **p<*0.05, ***p<*0.01, ****p<*0.001, *****p<*0.0001

**Figure S3. Identification of elevated CXCL9 expression in endothelial cells around the central vein within the lobule.**

(a) The RCI of the known chemokines associated with KC migration. The RCI for macrophage-recruiting chemokines were evaluated based on the ST data from both control and CHB mice at different time points to reveal their spatial expression pattern. (b) The representative immunofluorescent images of peri-portal (Zone 1) and peri-central (Zone 3) distribution and delineation of the region of interest (ROI) for DSP analysis within the FFPE sections of livers from both HBV transgenic and control mice. The antibodies for GS (green), F4/80 (red), and Syto13 (blue) were used to identify liver zonation with spatial information in IF assays for the subsequent transcriptome analysis of selected cells. The bar plot showed *Cxcl9* expression levels comparing between transgenic and control mice within Zone 1 and Zone 3. (c-d) The violin plot (c) and feature plot (d) of the expression of *Cxcl9* in all the identified cell types by scRNA-seq data. *CXCL9* was found to be exclusively expressed in endothelial cells. (e) Western blot analysis of CXCL9 in primary human liver sinusoid endothelial cells (HLSEC) co-cultured with tet^+^ or tet^-^ HepAD38 cells. (f) Western blot analysis of CXCL9 in HLSEC cells treated with si-CXCL9 for 72 hours was shown, and the result was quantified by grayscale value. (g) The representitive mIF images of the liver tissues from the control and CHB mice at 3-, 6-, and 12-months post infection by co-staining CXCL9, CXCR3, CD31 (endothelial cell marker), GS (peri-central marker), F4/80 (KC marker) and DAPI. CXCL9 was detected to be increased in CD31^+^ endothelial cells within the GS^+^ peri-central area with the progression of CHB infection. Data are shown as mean ± SEM; **p<*0.05, ***p<*0.01, ****p<*0.001, *****p<*0.0001

**Figure S4.** **Lipid metabolism disorders in KCs upon CHB infection.**

(a) Volcano plots of DEGs (derived from the above scRNA-seq analysis) in Kupffer cells of AAV-HBV mice at indicated time points. (b) M&T staining of liver sections from HBV transgenic mice with fibrosis. (c) GO analysis of DEGs in Kupffer cells of HBV transgenic mice based on DSP data. (d) Schematics of the co-culture system between HepAD38 cells and THP-1-derived macrophages. HepAD38/tet^+^ cells were deprived of tetracyline for four to six days to induce efficient HBV expression, followed by co-culture with THP-1-derived macrophages for another two days for subsequent analysis. (e) Western blotting of HBc subunits and HBc nucleocapsids (NC), and Southern blotting for HBV core DNAs in THP-derived macrophages co-cultured with HepAD38 cells (tet^-^ or tet^+^). HepAD38 cells were used as a positive control for HBV infection. (f) The representative IF images of the expression of LXRα in KCs of the livers isolated from the control and CHB mice at different time points. Anti-LXRα and anti-Clec4f antibodies were co-stained in the IF analysis. DAPI was used to stain the nucleus. The representative images of Con-27m group were same field as the representative images of Control-27m group in Figure 2g. (g) Quantitative analysis of LXRα expression in Kupffer cells (KCs) derived from the peri-portal and peri-central regions of control and CHB mice. The liver tissues were isolated from the control and CHB mice of different time points, followed by IF analysis via co-staining the anti-LXRα, anti-Clec4f, anti-GS, and anti-E-cadherin antibodies. Three fields of view on the representative images of IF were selected from the control and CHB mice for the counting of KCs. *p<0.05, **p<0.01, ***p<0.001, ****p<0.0001

**Figure S5. The pro-fibrosis/tumorigenesis effect of LMD macrophages and the identification of potential key regulators mediating CSC formation within the crosstalk of HepAD38/tet^-^ and LMD macrophages.**

(a) The enrichment of cancer hallmarks and oncogenic pathways in the hepatocytes of CHB mice at different hepatopathological stages. Standard GSEA, combined with MSigDB v7.5 collections hallmarks signatures (MH) analysis, was performed to explore the oncogenic pathways in hepatocytes based on the scRNA-seq data. (b) No direct inhibitory effect of GW3965 on the expression of Yamanaka factors in HepAD38 cells. LMD macrophages were withdrawed from their co-culture with HepAD38/tet^-^ and then GW3965 was added to the treated HepAD38/tet^-^ cells to see if there was any direct effect of GW3965 on the expression of pluripotency factors induced by LMD macrophages. The experiments were performed in triplicate (n = 3). (c) HepAD38 cells (tet-), pre-cocultured with THP-1 macrophages with or without LMD were collected and subjected to flow cytometry analysis of the well-established liver CSC markers (CD133, CD90). The percentages of CD133^+^ and CD90^+^ HepAD38 cells were significantly induced in HepAD38 cells cocultured with LMD THP-1 macrophages (n=3). (d) The mRNA expression of the fibrosis biomarkers (*TGF-β* and *MMP-2*) were evaluated in HBV^+^ HepAD38 cells treated by macrophages with or without LMD. The experiments were performed in triplicate (n = 3). (e) The reversal role of GW3965 in LMD macrophage-increased mRNA level of *TGF-β* and *MMP-2* in HBV^+^ HepAD38. The experiments were performed in triplicate (n = 3). (f) The workflow of Olink inflammation panel analysis for the supernatant derived from the co-culture system between HepAD38/tet^-^ and THP-1-derived macrophages (normal or LMD) with or without GW3965. Triplicate of supernatant from each group were subjected to Olink analysis. (g-h) The volcano plots of inflammatory DEPs obtained by Olink assay between the normal and LMD macrophage co-culture system (g), and between LMD macrophage co-culture system with and without GW3965 (h). (i) Protein-protein interaction assay (left) and correlation analysis (right) of the identified potential inflammatory mediators within the crosstalk. (j) The heatmap of the expression of the identified DEPs was displayed. (k) Normalized protein expression level of the identified inflammatory candidates obtained from the Olink data. Data shown are mean ± SEM; **p<*0.05, ***p<*0.01, ****p<*0.001, *****p<*0.0001

**Figure S6.** **Dysregulation of LXRα in Kupffer cells within HBV-associated HCC patients.**

(a) The basic information, tumor characteristics, and pathologic data of HBV-associated HCC patients. The counts of LXRα^+^ KCs within the paied normal, tumor adjacent, and tumor tissues of the patients are also shown in the chart.
