# Supplementary material for: CHB‐Induced Immune Zonation Chaos Elicited LXRα‐mediated Lipid Metabolism Disorders in Kupffer Cells to Induce Cancer Stem Cell Formation
Source: Adv Sci (Weinh). 2025 Oct 30;13(10):e10275. doi: 10.1002/advs.202510275 (PMC12915127; doi:10.1002/advs.202510275)
Supplement: Supplementary file 2 — Supporting Tables [file ADVS-13-e10275-s001.docx]

**CTAT methods**

- 1. **Antibodies**

| **Name** | **Citation** | **Supplier** | **Cat no.** | **Clone no.** |
| --- | --- | --- | --- | --- |
| Rb polyclonal anti-GS | RRID:AB_2247588 | Abcam | ab73593 | N/A |
| Ms monoclonal anti-HBcAg | RRID:AB_306686 | Abcam | ab8639 | 10E11 |
| Rb polyclonal anti-HBsAg | RRID:AB_1209673 | Abcam | ab68520 | N/A |
| Rb monoclonal anti-F4/80 | RRID:AB_2799771 | Cell Signaling Technology | 70076 | D2S9R |
| Rb monoclonal anti-CD31 | RRID:AB_2722705 | Cell Signaling Technology | 77699 | D8V9E |
| Rb polyclonal anti-E-cadherin | RRID: AB_3661873 | Sino Biological | 50671-RP02 | N/A |
| Rb polyclonal anti-LXRα | RRID:AB_10640525 | Proteintech | 14351-1-AP | N/A |
| Rb polyclonal anti-CXCL9 | RRID:AB_2879086 | Proteintech | 22355-1-AP | N/A |
| Rb polyclonal anti-CXCR3 | RRID:AB_2880623 | Proteintech | 26756-1-AP | N/A |
| Rb polyclonal anti-NANOG | RRID:AB_1607719 | Proteintech | 14295-1-AP | N/A |
| Rb polyclonal anti-ASGR1 | RRID:AB_2059675 | Proteintech | 11739-1-AP | N/A |
| Rb polyclonal anti-BRCA1 | RRID:AB_2879090 | Proteintech | 22362-1-AP | N/A |
| Rb polyclonal anti-BARD1 | RRID:AB_2879190 | Proteintech | 22964-1-AP | N/A |
| Rb polyclonal anti-LIN28 | RRID:AB_2135039 | Proteintech | 11724-1-AP | N/A |
| Rb polyclonal anti-OCT4 | RRID:AB_2167545 | Proteintech | 11263-1-AP | N/A |
| Rb polyclonal anti-STAT3 | RRID:AB_2302876 | Proteintech | 10253-2-AP | N/A |
| Rb polyclonal anti-WNT3A | RRID:AB_2918108 | Proteintech | 26744-1-AP | N/A |
| Rb monoclonal anti-ABCA1 | RRID: AB_3661872 | Cell Signaling Technology | 96292 | E7X5G |
| Rb monoclonal anti-p-STAT3 | RRID:AB_2491009 | Cell Signaling Technology | 9145 | D3A7 |
| Rb monoclonal anti-p-AKT | RRID:AB_2315049 | Cell Signaling Technology | 4060 | D9E |
| Rb monoclonal anti-AKT | RRID:AB_915783 | Cell Signaling Technology | 4691 | C67E7 |
| Ms monoclonal anti-HBc antigen | RRID: AB_3661876 | Tokyo Future Style | 2AHC24 | T2221 |
| Alexa fluor 700 anti-mouse CD45.2 | RRID:AB_493731 | Biolegened | 109822 | 104 |
| Brilliant Violet 421™ anti-mouse CD31 | RRID:AB_2562186 | Biolegened | 102423 | 390 |
| APC anti-mouse CD204 | RRID:AB_2892311 | Biolegened | 154711 | 1F8C33 |
| PE/Cyanine7 anti-mouse CD117 (c-kit) | RRID:AB_313222 | Biolegened | 105813 | 2B8 |
| PE anti-mouse CXCL9 (MIG) | RRID:AB_2245490 | Biolegened | 515603 | MIG-2F5.5 |
| Goat polyclonal anti-CCL19 | RRID:AB_2605836 | Thermo Fisher | PA5-46940 | N/A |
| Ms monoclonal anti-CXCL10 | RRID:AB_2609776 | Thermo Fisher | MA5-23819 | 33036 |
| Ms monoclonal anti-CXCL11 | RRID:AB_2610462 | Thermo Fisher | MA5-23761 | 87328 |
| Ms monoclonal anti-GS-AF532 | RRID:AB_3361437 | Novus Biologicals | NBP2-70834AF532 | OTI1F4 |
| Rat monoclonal anti-F4/80-AF647 | RRID:AB_2810932 | Abcam | ab204467 | F4/80 |

- 1. **Cell lines**

| **Name** | **Citation** | **Supplier** | **Cat no.** | **Passage no.** | **Authentication test method** |
| --- | --- | --- | --- | --- | --- |
| THP-1 | RRID:CVCL_0006 | Cell Resource Center,Institute of Basic Medical Sciences | 1101HUM-PUMC000057 | 5 | N/A |
| HLSEC | RRID:CVCL_QY34 | Meisen Cell Technology | CTCC-001-0418 | 2 | N/A |
| HepAD38 | RRID:CVCL_M177 | Jieliang Chen Lab | N/A | 5 | N/A |

- 1. **Organisms**

| **Name** | **Citation** | **Supplier** | **Strain** | **Sex** | **Age** | **Overall n number** |
| --- | --- | --- | --- | --- | --- | --- |
| Mouse | RRID:MGI:3028467 | Vital River Laboratory Animal Technology | C57BL/6J | male | 4-6 weeks | 100 |
| Hepatitis B virus | RRID:NCBITaxon_928302 | Packgene | AAV8-HBV-D genotype, ayw | N/A | N/A | N/A |
| Control virus | N/A | vigene | AAV8-EGFP | N/A | N/A | N/A |

- 1. **Sequence based reagents**

| **Name** | **Sequence** | **Supplier** |
| --- | --- | --- |
| si*CXCL9*-1 target sequence | 5’- CCA AGG GAC TAT CCA CCT A -3’ | RiboBio |
| si*CXCL9*-2 target sequence | 5’- GTT CGA AAA TCT CAA CGT T -3’ | RiboBio |
| si*CXCL9*-3 target sequence | 5’- GGA GTT CAA ACA TGT CTA A -3’ | RiboBio |
| si*ASGR1*-1 target sequence | 5’- TGC TCC ACG TGA AGC AGT T -3’ | RiboBio |
| si*ASGR1*-2 target sequence | 5’- TGA CCA CCA TCA GCT CAG A -3’ | RiboBio |
| si*ASGR1*-3 target sequence | 5’- GAG GCA ATG TGG GAA GAA A -3’ | RiboBio |
| si*STAT3*-1 target sequence | 5’- GGC GTC CAG TTC ACT ACT A -3’ | RiboBio |
| si*STAT3*-2 target sequence | 5’- AGA CCC GTC AAC AAA TTA A -3’ | RiboBio |
| si*STAT3*-3 target sequence | 5’- CAT CGA GCA GCT GAC TACA -3’ | RiboBio |
| si*AKT*-1 sense | 5’- GCU ACU UCC UCC UCA AGA ATT -3’ | GenePharma |
| si*AKT*-1 antisense | 5’- UUC UUG AGG AGG AAG UAG CTT -3’ | GenePharma |
| si*AKT*-2 sense | 5’- CCA UGA AGA UCC UCA AGA ATT -3’ | GenePharma |
| si*AKT*-2 antisense | 5’- UUC UUG AGG AUC UUC AUG GTT -3’ | GenePharma |
| si*AKT*-3 sense | 5’- CGG AGA AGA ACG UGG UGU ATT -3’ | GenePharma |
| si*AKT*-3 antisense | 5’- UAC ACC ACG UUC UUC UCC GTT -3’ | GenePharma |
| has-miR-155-5p inhibitor | 5’- AAC CCC UAU CAC GAU UAG CAU UAA -3’ | GenePharma |
| has-miR-206 inhibitor | 5’- CCA CAC ACU UCC UUA CAU UCC A -3’ | GenePharma |
| miR-inhibitor N.C. | 5’- CAG UAC UUU UGU GUA GUA CAA -3’ | GenePharma |
| *NANOG* | \| F: 5’- CAC CTA TGC CTG TGA TTT G -3’ \| \| --- \| \| R: 5’- TTG TTT GCC TTT GGG AC -3’ \| | This paper |
| *OCT4* | \| F: 5’- TGA GGG CGA AGC AGG AG -3’ \| \| --- \| \| R: 5’- TCA AAG CGG CAG ATG GTC -3’ \| | This paper |
| *cMYC* | \| F: 5’- GAG GAG GAA CAA GAA GAT GAG G -3’ \| \| --- \| \| R: 5’- AGG ACC AGT GGG CTG TGA GG -3’ \| | This paper |
| *KLF4* | \| F: 5’- CCA GAG GAG CCC AAG CCA AAG -3’ \| \| --- \| \| R: 5’- TCC ACA GCC GTC CCA GTC A -3’ \| | This paper |
| *LIN28A* | \| F: 5’- CCA GTG GAT GTC TTT GTG CAC C -3’ \| \| --- \| \| R: 5’- GTG ACA CGG ATG GAT TCC AGA C -3’ \| | This paper |
| *ACTIN* | \| F: 5’- CAC CAT TGG CAA TGA GCG GTT C -3’ \| \| --- \| \| R: 5’- AGG TCT TTG CGG ATG TCC ACG T -3’ \| | This paper |
| *MMP2* | \| F: 5’- GGC CAG ATC CTG TCC AAG C -3’ \| \| --- \| \| R: 5’- GTG GGT TTC CAC CAT TAG CAC -3’ \| | This paper |
| *TGFB1* | F: 5’- GGC CAG ATC CTG TCC AAG C -3’  R: 5’- GTG GGT TTC CAC CAT TAG CAC -3’ | This paper |
| *CXCR3* | F: 5’- CCA CCT AGC TGT AGC AGA CAC -3’  R: 5’- AGG GCT CCT GCG TAG AAG TT -3’ | This paper |
| *Nr1h3* | F: 5’- CTC AAT GCC TGA TGT TTC TCC T -3’  R: 5’- TCC AAC CCT ATC CCT AAA GCA A -3’ | This paper |
| *Abca1* | \| F: 5’- AAA ACC GCA GAC ATC CTT CAG -3’ \| \| --- \| \| R: 5’- CAT ACC GAA ACT CGT TCA CCC -3’ \| | This paper |
| *Abcg1* | \| F: 5’- CTT TCC TAC TCT GTA CCC GAG G -3’ \| \| --- \| \| R: 5’- CGG GGC ATT CCA TTG ATA AGG -3’ \| | This paper |
| *Apoa1* | \| F: 5’- GGC ACG TAT GGC AGC AAG AT -3’ \| \| --- \| \| R: 5’- CCA AGG AGG AGG ATT CAA ACT G -3’ \| | This paper |
| *Apoe* | F: 5’- CTG ACA GGA TGC CTA GCC G -3’  R: 5’- CGC AGG TAA TCC CAG AAG C -3’ | This paper |
| *Col1a1* | F: 5’- GCT CCT CTT AGG GGC CAC T -3’  R: 5’- CCA CGT CTC ACC ATT GGG G -3’ | This paper |
| *Mmp2* | F: 5’- CAA GTT CCC CGG CGA TGT C -3’  R: 5’- TTC TGG TCA AGG TCA CCT GTC -3’ | This paper |
| *Cxcr3* | F: 5’- TAC CTT GAG GTT AGT GAA CGT CA -3’  R: 5’- CGC TCT CGT TTT CCC CAT AAT C -3’ | This paper |
| *Actin* | F: 5’- CGT TGA CAT CCG TAA AGA CCT C -3’  R: 5’- ACA GAG TAC TTG CGC TCA GGA G -3’ | This paper |

- 1. **Biological samples**

| **Description** | **Source** | **Identifier** |
| --- | --- | --- |
| HBV-related HCC Tissue chip | Raisedragon’S | N/A |

- 1. **Deposited data**

| **Name of repository** | **Identifier** | **Link** |
| --- | --- | --- |
| Raw and analyzed data | This paper; China National GeneBank DataBase (CNGBdb) | https://db.cngb.org/ (CNP0006059) |
| HBV genotype D | GenBank: AJ344117.1 | https://www.ncbi.nlm.nih.gov/nuccore/AJ344117.1 |
| Code for analysis | This paper; Github | https://github.com/QingyuLiaib/HBV_scRNA |

- 1. **Software**

| **Software name** | **Manufacturer** | **Version** |
| --- | --- | --- |
| ImageJ | Schneider et al. | https://imagej.nih.gov/ij/ |
| Graphpad | GraphPad Software | https://www.graphpad.com/ |
| Cytoscape | Shannon P et al. | https://cytoscape.org / |
| Clusterprofiler | Yu G et al. | https://www.bioconductor.org/packages/release/bioc/html/clusterProfiler.html |
| Seurat | Satija et al. | https://github.com/satijalab/seurat |
| RCTD | Dylan et al. | https://github.com/dmcable/spacexr |

- 1. **Other (e.g. drugs, proteins, vectors etc.)**

| 7500 Real-Time PCR System | Applied Biosystems | RRID:SCR_018051 |
| --- | --- | --- |
| EVOSTM XL Core | Themo Fisher | Cat#AMEX1000 |
| TECAN Infinite 200 | TECAN | TECAN Infinite 200 |
| FBS | Gibco | Cat#10099141C |
| Trypsin-EDTA | Sigma | Cat#T4049 |
| PBS | Gibco | Cat#20012027 |
| DMEM | Gibco | Cat#11320033 |
| RPMI-1640 | Gibco | Cat#11875093 |
| Tetracycline | M&C GENE TECHNOLOGY | Cat#MA013 |
| G418 | MedChemExpress | Cat#HY-17561 |
| BSA | Sigma | Cat#V900933 |
| PMA | Sigma | Cat#P1585 |
| N2 | Themo Fisher | Cat#C046 |
| B27 | Themo Fisher | Cat#17502048 |
| EGF | Novoprotein | Cat#C029 |
| FGFb | Novoprotein | Cat#C046 |
| MG132 | MedChemExpress | Cat#HY-13259 |
| T0901317 | MedChemExpress | Cat#HY-10626 |
| GW3965 | MedChemExpress | Cat#HY-10627A |
| HBsAg detection kit | Darui Bio Technology | Cat#DR-ID-B001 |
| HBeAg detection kit | Darui Bio Technology | Cat#DR-ID-B003 |
| HBV DNA detection kit | Sansure Biotech | Cat#SX-fsstHBV01 |
| Mouse αFP ELISA kit | Elabscience | Cat#E-EL-M2405c |
| liver perfusion kit | Liver Biotechnology | Cat#LV-PHIK001-5T |
| MACS cell debris removal kit | Miltenyi Biotec | Cat#130-109-398 |
| MACS dead cell removal kit | Miltenyi Biotec | Cat#130-090-101 |
| Liver dissociation kit | Miltenyi Biotec | Cat#130-105-807 |
| AO/PI | CellAegis Devices | Cat#F23001 |
| OctoMACS Separator | Miltenyi Biotec | Cat#130-042-109 |
| gentle MACS C tube | Miltenyi Biotec | Cat#130-093-237 |
| 40/70/100 μm smart strainer | Miltenyi Biotec | Cat#130-098-462 |
